# Supramolecular Combination Cancer Therapy Based on Macrocyclic Supramolecular Materials

**DOI:** 10.3390/polym14224855

**Published:** 2022-11-11

**Authors:** Yilin Li, Yuteng Su, Zhaoxiang Li, Yueyue Chen

**Affiliations:** Department of Toxicology and Sanitary Chemistry, School of Public Health, Beijing Key Laboratory of Environment Toxicology, Capital Medical University, Beijing 100069, China

**Keywords:** multivalent supramolecular assembly, cucurbituril, cyclodextrin, pillararene, combination therapy

## Abstract

Supramolecular combination therapy adopts supramolecular materials to design intelligent drug delivery systems with different strategies for cancer treatments. Thereinto, macrocyclic supramolecular materials play a crucial role in encapsulating anticancer drugs to improve anticancer efficiency and decrease toxicity towards normal tissue by host–guest interaction. In general, chemotherapy is still common therapy for solid tumors in clinics. However, supramolecular combination therapy can overcome the limitations of the traditional single-drug chemotherapy in the laboratory findings. In this review, we summarized the combination chemotherapy, photothermal chemotherapy, and gene chemotherapy based on macrocyclic supramolecular materials. Finally, the application prospects in supramolecular combination therapy are discussed.

## 1. Introduction

Cancer is one of the most concerning public health problems. Although chemotherapy is the mainstream mean of cancer treatment, some chemotherapeutic drugs have disadvantages, such as poor water solubility [1], strong systemic cyto-toxicity [2], and a lack of specificity [3]. These limitations greatly reduce the treatment efficiency. To address this problem, scientists are currently designing new drug delivery systems based on polymers [4], liposomes [5], and inorganic nanomaterials [6,7]. Combining supramolecular macrocyclic materials with drugs, supramolecular anti-cancer drugs are constructed through host–guest interactions [8,9]. Supramolecular macrocyclic host–guest complex can significantly lessen the toxicity of chemical medications to healthy cells [10], and at the same time, selective drug administration can be accomplished through a stimulus response [11,12]. Besides, using supramolecular macrocyclic host–guest complex as block connections will greatly simplify the preparation process [13]. The supramolecular chemotherapy combined with other therapies can enhance the anti-cancer effectiveness of drugs and reduce the negative effects of conventional chemotherapy ultimately [14,15]. Supramolecular macrocyclic materials contain cyclodextrin [16], pillararene [17,18], cucurbituril [19], and metal coordination complexes [20]. Comparing the features of these four nanomaterials, they all realize their drug delivery function through host–guest interaction. Among the macrocyclic molecules, cyclodex-trin has the smallest cavity, pillararene is the second, and cucurbituril is the largest. Since metal coordination can adjust its synthesis method to fit the size of the guest molecule, it can be used for larger drug molecule delivery [21]. This paper reviews the research progress of combination therapy between supramolecular chemotherapy and other therapies based on supramolecular macrocyclic materials.

## 2. Supramolecular Chemotherapy and Combination Therapy Based on Cyclodextrins

Cyclo[n]dextrin (CDn) is a series of cyclic oligomers with 6–12 glucopyranose units. As a kind of macrocyclic molecule with hydrophobic cavities structure, cyclodextrin can trap or encapsulate appropriately sized guest species with various binding affinities through van der Waals interactions and hydrophobic force in aqueous media. In other words, cyclodextrin can act as a host cavity to encapsulate a great number of anti-cancer drugs. Due to its good water solubility, low toxicity, and biocompatibility, cyclodextrin has been used to improve the solubility or stability of various chemotherapeutic drugs in recent years, and it is considered as a promising material among various nanocarriers.

### 2.1. Supramolecular Chemotherapy Based on Cyclodextrins

In the fabrication of supramolecular chemotherapeutic agents, hydrophobic anti-cancer medications and cyclodextrins can form host–guest inclusion complexes. It upgrades the solubility, stability, and safety of the medications. It is favorable to the improvement of their availability in biological systems. Based on the remarkable stability, admirable biocompatibility, and non-toxicity toward human normal cells, its structure is susceptible to the parental modification of cyclodextrins. Numerous cyclodextrin derivatives have been acknowledged as platforms because they are optional for the host–guest molecular recognition-based production of supramolecular chemotherapeutic drugs.

Lin and co-workers fabricated a POSS-based supramolecular AD-POSS-(sulfobetaine)7/CD-PLLA zwitterionic complex via host–guest interaction, which encapsulated anti-cancer drug models (DOX), and had shown potential application value in tumor inhibition [22]. In previous studies, scientists had used the host–guest interaction between cyclodextrin and polyethylene glycol to prepare cyclodextrin-based supramolecular polymers, studied their loading effects on drugs, and found that they were of great significance in the treatment of tumors. Cheng and co-workers constructed a temperature-sensitive β-CD-g-(PNIPAAm-b-POEGA)x drug complex nanoparticles, which had good biocompatibility and effectively inhibited the proliferation and differentiation of MDR1-resistant tumor cells [23]. Moreover, Song and co-workers synthesized PNIPAAm with a β-cyclodextrin core and an Ad-PEG polymer. It produced a stronger cellular absorption and anti-cancer activity than free DOX controls, even in cancer cells that were resistant to multiple drugs [24]. Yu and co-workers designed a drug delivery system via non-covalent host–guest interaction between HA-β-CD and 4-arm-PEG-Ad, which enhanced the efficacy of chemotherapy drugs [25], and both of these two supramolecular systems released drugs through the dissolution of hydrogels.

**Figure 1:**
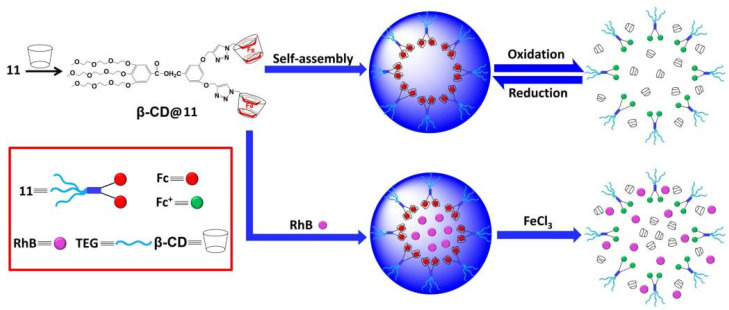
The Janus supramolecule β-CD@11’s synthesis, redox-responsive self-assembly, drug loading, and oxidation-triggered release are shown schematically (reproduced with permission of Elsevier from ref. [28]).

With the development of modern medicine, designing multifunctional cyclodextrin nanoparticles that can react to outside stimuli and mimic the responsiveness and improving the adaptability of biological materials has grown to be a popular issue in nanotechnology [26]. Shi and co-workers constructed supramolecular amphiphiles via host–guest interactions between Fc-AcMH and MH4-β-CD, and AcMH facilitated pH-mediated drug release. The nanoparticles could be disintegrated at pH 4 [27]. Mu and co-workers synthesized a new redox supramolecular Janus device via β-cyclodextrin and 2-fold ferrocene host–guest interactions, which induced the release of the cargo through the oxidation of the ferrocene group (Figure 1) [28].

**Figure 2:**
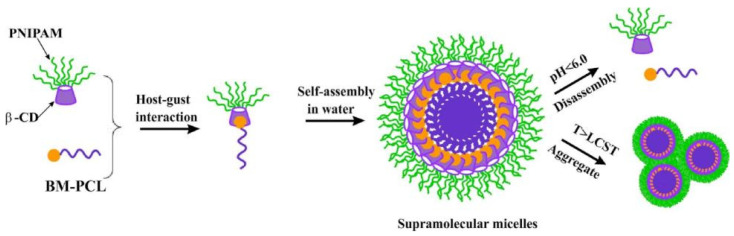
Diagram illustrating the creation of temperature- and pH-sensitive supramolecular micelles from the star polymer β-CD-PNIPAM (reproduced with permission of Elsevier from ref. [30]).

Due to its unique advantages in controlled drug release, pH-sensitive nanocarriers have drawn extensive attention. Zhang and co-workers developed a supramolecular polymer β-CD-g-PDMAEMA@Azo-PCL via the host–guest interaction between β-CD-g-PDMAEMA and Azo-PCL, which exhibited drug release characteristics and regulated to light and pH [29]. Zhou and co-workers constructed temperature/pH-sensitive supramolecular micelle by using star polymer β-CD-PNIPAM and BM-PCL via host–guest reversible recognition (Figure 2) [30]. Gao and co-workers developed poly(cyclodextrin)-containing block copolymer PEG-PCD with benzimidazole-modified poly(caprolactone) to form pH-sensitive complex polymer micelles (BM-PCL), and the release of drugs was accelerated as the pH reduced from 7.0 to 2.0 and the temperature increased from 25 to 37 °C [31].

In addition, previous studies have covered many supramolecular compounds formed by the interaction between cyclodextrin and other small molecule drugs or polymers. Bai and co-workers studied the creation of supramolecular self-assemblies and their behavior via self-assembly between HA-β-CD and drug–drug conjugates (curcumin-oxoplatin, Cur-Pt) [32]. Xing and co-workers assembled PEGylated cyclodextrin into a supramolecular polymer together with SS-DOX formed by two doxorubicin linked by the disulfide bond [33]. Yang and co-workers synthesized the carboxyethyl hydroxyethyl cellulose-grafted with adamantane and the β-cyclodextrin-grafted glycerol ethoxylate [34]. Torchio and co-workers developed SM hydrogels and high-molar mass amphiphilic PEUs, respectively, based on α-cyclodextrin and Poloxamer^®^407 [35]. Yu and co-workers produced a theranostic supramolecular polymer in a creative way. In their work, camptothecin was considered as the guest and attached to the host compound β-cyclodextrin through a glutathione-cleavable disulfide bond [36]. Liu and co-workers first reported a naturally occurring supramolecular polymer micelle, which was formed by single-stranded chains of curdlan and β-cyclodextrins, and it encapsulated CPT as a model drug [37]. Owing to the disulfide bonds of drug dimers, these supramolecular micelles were dissociated, and the drugs were recovered and released from cavities of cyclodextrins because of dynamic equilibrium and hydrophilicity changes.

### 2.2. Supramolecular Combination Chemotherapy Based on Cyclodextrins

Combination chemotherapy refers to the use of two or more chemotherapy drugs for chemotherapy. Selecting chemotherapy drugs with different mechanisms of action can improve the anti-cancer efficacy and further prolong the life of patients. However, the negative effects of combination chemotherapy are relatively obvious, such as bone marrow suppression [38], gastrointestinal reactions [39], and hepatic and renal toxicity [40]. Supramolecular combination chemotherapy achieves precise delivery and controlled release of combined drugs through the efficient combination of chemotherapy drugs and biological macromolecular drugs, reducing drug release outside the target organs, which had shown low toxicity and high efficacy in varieties of past studies.

Li and co-workers constructed nanoparticle polymers for combined chemotherapy and tailored drug delivery via interactions between the three components’ hosts and guests. As the backbone of the nanoparticles, β-cyclodextrin polymer (poly-β-CD) combined two anti-tumor drugs—doxorubicin (DOX) and docetaxel (DTX)—and then formed self-assembled supramolecular nanoparticles (SSNPs). As identification elements, the aptamers were then modified on the surface of SSNPs. Labeled with adamantane and fluorescein (Ad-aptamer-FAM), aptamers made SSNPs capable of targeting. Finally, target self-assembled supramolecular nanoparticles (T-SSNPs) were constructed. MTS experimental data showed that T-SSNPs had significant cytotoxicity to target cells and had a positive synergistic impact on combination chemotherapy, making it an efficient and simple medication delivery device for combination chemotherapy and targeted drug delivery [41].

Huang and co-workers mixed α-cyclodextrin aqueous solution with MPEG-PCL micelles, and prepared supramolecular hydrogels via the host–guest recognition. The co-transfer of dexamethasone sodium phosphate (Dexp) and Avastin (Ava) could be realized by dissolving in the above solution, and the designed hydrogels controlled drug release by adjusting the amounts of 4-armPEG-Ad. The supramolecular hydrogel showed good cytocompatibility and almost no cytotoxicity in both cell experiments and rat models. Compared with Ava drugs alone, Ava hydrogel drugs had a longer retention time in organisms and higher bioavailability, resulting in better therapeutic effects [42].

### 2.3. Supramolecular Chemotherapy Combined with Photothermal Therapy Based on Cyclodextrins

Photothermal therapy is a kind of photochemical reaction caused by the excitation of photosensitizer through light energy, the basis action of which is the photodynamic effect. The photosensitization reaction involves oxygen molecules with biological effects, which uses photosensitizers to produce a significant amount of reactive oxygen species (ROS) under specific wavelength laser irradiation, and it exerts anti-tumor effects by activating the mitochondrial and death receptor pathways of apoptosis and participating in autophagic cell death. As an emerging anti-tumor therapy, photothermal therapy has the advantages of weak invasiveness, high efficiency, low toxicity, and rare side effects. It is now mostly used in the therapeutic schedule of breast cancer [43,44], laryngeal cancer [45], and oral squamous cell carcinoma [46]. It has been shown that the co-administration of chemotherapeutic medicines and photosensitizers by using a nano-delivery system is expected to bring out the combined anti-tumor effect of photodynamic therapy and chemotherapy, thus making up for the shortcomings in the application of single treatment modality. Photothermal nanomaterials can not only improve the stability, bioavailability, and targeting of the treatment [47], but aid in therapeutic bio-imaging, which has shown unique advantages in the field of tumor treatment.

**Figure 3:**
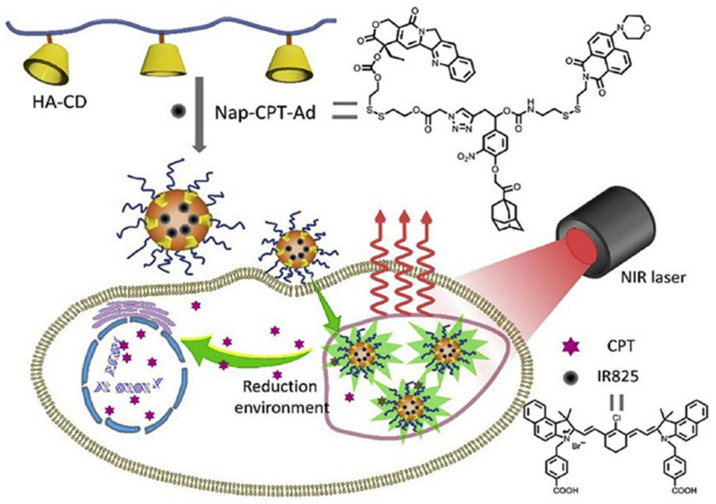
A reduction-sensitive supramolecular polymeric drug delivery system (reproduced with permission of Elsevier from ref. [48]).

A combinational photothermal chemotherapy approach for cancer was created by Zhang and co-workers, which was reduction-sensitive. The near-infrared (NIR) absorbing dye IR825 was loaded into the multifunctional system in the experiment using the supramolecular self-assembly approach. Hyaluronic acid was functionalized by hydrophilic cyclodextrin, which also ensured the nanoparticles’ good colloidal biocompatibility and stability. The camptothecin/dye conjugate’s embedded disulfide link was broken in a reducing environment, releasing the conjugated drug and restoring fluorescence emission instantly. The nano platform was an effective device for photothermal therapy due to the effective conversion from the absorbed light into local heat by dye IR825. It demonstrated that CPT-HA@IR825 nanoparticles can kill HeLa, MCF-7, and U14 cancer cells by confocal microscopy photos and a vivo investigation when exposed to laser irradiation. This research provided a dual delivery strategy for maximizing the effectiveness of combination photothermal chemotherapy therapy for cancer (Figure 3) [48].

Liu and co-workers developed an injectable nanocomposite hydrogel made of paclitaxel-loaded nanoparticles and gold nanorods, and used methoxy poly(ethylene glycol)-*b*-poly(ε-caprolactone-*co*-1,4,8-trioxa[4.6]spiro-9-undecanone) (mPECT) diblock copolymer to prepare mPECT-modified gold nanorods (AuNR-PECT) and paclitaxel-loaded mPECT nanoparticles (PTX/mPECT NPs). After that, ^AuNR/PTX^mPECT^gel^, the injectable nanocomposite hydrogel mentioned above was created by the host–guest interaction between α-cyclodextrin, PTX/mPECT NPs, and AuNR-PEC. In vivo and in vitro experiments confirmed that the injectable nano-composite hydrogel had a significant near-infrared radiation photothermal effect, which effectively inhibited the proliferation and differentiation of tumor cells and enhanced the curative effect of chemo-photothermal synergetic cancer therapy [49].

By the host–guest self-assembly of α-cyclodextrin and photothermal conjugated polymers, Liu and colleagues created an injectable thermosensitive photothermal-network hydrogel (PNT-gel). The great photothermal conversion efficiency and photothermal stability of PNT-gel were ensured by the conjugated polymer backbones’ direct conversion of light energy into heat. At the same time, the hydrogel’s shear-thinning injectability, photothermally-driven, and reversible gel-sol conversion were realized by the moderate host–guest assembly, which also brought remote control and on-demand release of drugs to pass. What is more, the presence of hydrogels prolonged retention time, which made it possible for infrared therapy to be repeated [50].

**Figure 4:**
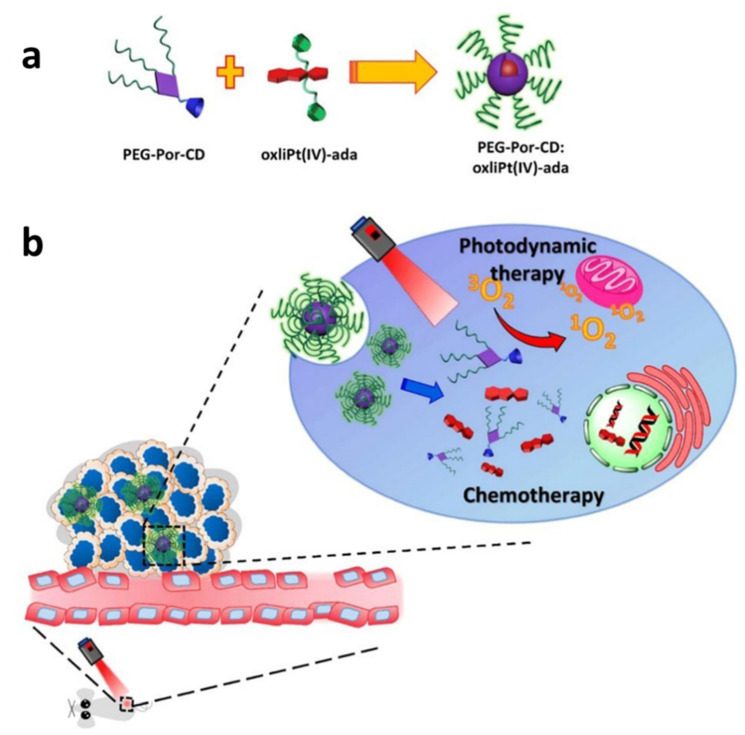
(**a**) OxliPt(IV)-ADA nanoparticle: schematic illustration for the self-assembled formation of PEG-Por-CD and (**b**) its application in combination with chemotherapy and photodynamic therapy for cancer (reproduced with permission of American Chemical Society from ref. [51]).

Based on host–guest interaction, Lim and co-workers designed a kind of nanocarrier, assembled (PEG-Por-CD: oxaliplatin-ADA) to achieve stimulus-responsive combination therapy. As porphyrin and oxaliplatin have good spatial control of host–guest molecular binding ratio, researchers modified porphyrin and oxaliplatin with ß-cyclodextrin and adamantane, respectively, to prepare amphiphilic host–guest complexes and then self-assembled into therapeutic nanoparticles. Due to its unique nanostructure and combination therapy advantages, it was easier to construct PEG-Por-CD: oxaliplatin-ADA nanoparticles with optimized load ratio. The nanoparticle complex had good colloid stability and was decomposed in a reductive environment to liberate active pharmaceutical ingredients. Confocal imaging showed that under 630 nm light irradiation, PEG-Por-CD: oxaliplatin-ADA nanoparticles efficiently assembled in cells and created reactive oxygen species in vitro. Compared to monotherapy, PEG-Por-CD: oxaliplatin-ADA nanoparticles showed a 3-fold increase in cytotoxicity and a 2-fold increase in apoptosis. In vivo experiments on mice confirmed that nanoparticles effectively inhibited tumor growth without systemic toxicity and had good biocompatibility. PEG-Por-CD: oxaliplatin-ada nanoparticles showed promising therapeutic prospects in vitro and in vivo (Figure 4) [51].

In photodynamic therapy, supramolecular chemistry can not only carry drugs, but can also alter the chemical and physical characteristics of medications, thus enhancing the efficacy of photodynamic therapy. Through the host–guest inclusion interaction between bifunctional adamantane-conjugated porphyrin (TPP-Ad(2)) and cyclodextrin dimer (CD2), Jia and co-workers were able to create a linear alternating supramolecular polymer. Via steric hindrance, the supramolecular alternating structure of CD/TPP prevented PSs from aggregating, and enhanced the photophysical characteristics. Additionally, it improved the PSs’ water solubility to bring on by cyclodextrin moieties. This linear alternating supramolecular polymer (TPP-Ad(2)/CD2) created a nanoplatform that dramatically improved photodynamic treatment (PDT) [52].

**Figure 5:**
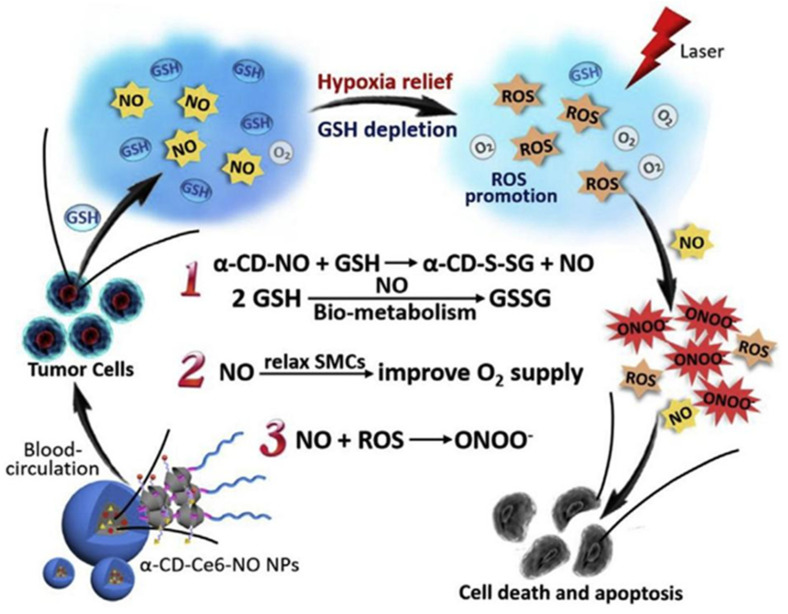
GSH-sensitive supramolecular nitric oxide (NO) nanogenerator (reproduced with permission of Elsevier from ref. [53]).

For instance, the conditions of a hypoxic environment and high-level intracellular glutathione (GSH) imposed restrictions on the production of ROS in the solid tumor microenvironment, which was produced when photo-activated photosensitizers reacted with the oxygen in the environment to cause cell death, necrosis, and tissue destruction in PDT. Utilizing the multifunctional NO as an all-arounder to enhance the therapeutic effectiveness of PDT, Deng and co-workers presented a novel complementary method to address the issue. In their study, scientists introduced a kind of supramolecular nitric oxide (NO) nanogenerator which was sensitive to GSH specializing in enhancing photodynamic therapy. The GSH-sensitive NO conveyor system was invented in the study by conjugating S-nitrosothiol to cyclodextrin, and the supramolecular nanocarrier CD-Ce6-NO nanoparticle was created by a host–guest interaction between prodrugs based on cyclodextrin and poly(ethylene glycol). The three points below served as the foundation for photodynamic therapy’s benefits. Initially, the GSH-freed NO exhibited the splendid ability to remove GSH. Furthermore, the SMC relaxation and increased blood flow caused by NO reduced the hypoxia at tumor locations. Continuous generation of the RNS by GSH-released NO and light-triggered ROS increased the biocidal action (Figure 5) [53].

Moreover, photodynamic therapy exerted an eminent implication on sterilizing non-tumor disease, for instance, biofilm sterilization. Han and co-workers investigated MMP-sensitive supramolecular nanoparticles to promote a photodynamic anti-bacterial action against bacterial keratitis linked with biofilms. Adamantane-encapsulated MMP-9-sensitive peptides and chlorin e6 conjugated β-cyclodextrin prodrug (β-CD-Ce6) served as the host and guest, respectively, in the study’s MMP-S NPs (Ad). In the keratitis microenvironment, overexpressed MMP-9 caused the protective EEEEEE peptide shell of MMP-SNPs to be removed. This exposed cationic peptides and allowed MMP-S NPs to penetrate and accumulate in the biofilms as well as bind to *P. aeruginosa*, which ultimately increased PDT’s antibacterial effectiveness. This exploration gave a feasible anti-bacterial solution to stifle bacterial keratitis by effectively wiping out irresistible microorganisms and eliminating bacterial biofilms in the cornea [54].

**Figure 6:**
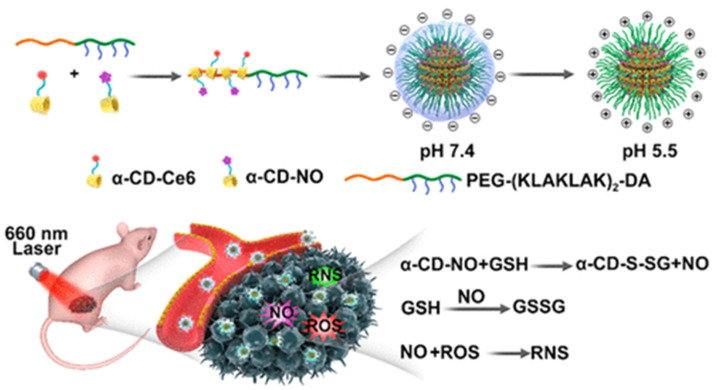
A surface charge switchable supramolecular nanocarrier (reproduced with permission of American Chemical Society from ref. [55]).

Nitric oxide (NO) and photodynamic eradication of the methicillin-resistant Staphylococcus aureus (MRSA) biofilm were successful thanks to Hu and co-workers’ development of a surface charge switchable supramolecular nanocarrier with penetration into an acidic biofilm which was closely correlated with pH. The pH-sensitive poly (ethylene glycol) (PEG) block polypeptide copolymer (PEG-(KLAKLAK)2-DA) was used as a host to integrate the GSH-sensitive cyclodextrin affiliated nitric oxide (NO) prodrug and chlorin e6 (Ce6) prodrug into supramolecular nanocarrier α-CD-Ce6-NO-DA. After infiltration of the biofilm, the GSH-triggered NO was released, resulting in a significant bactericidal effect and consumption of GSH. Simultaneously, reactive nitrogen species (RNS) were created when NO reacted with reactive oxygen species (ROS). These factors synergistically enhanced PDT efficiency. By utilizing the advantages listed above, the novel tactic established in this work may provide excellent opportunities to combat biofilm infections (Figure 6) [55].

### 2.4. Supramolecular Chemotherapy Combined with Gene Therapy Based on Cyclodextrins

Gene therapy for cancer uses vectors to transfer exogenous genes into patients through transgenic technology. With the emergence of a variety of biotechnology such as second-generation sequencing and gene editing, gene therapy is evolving and improving, and has achieved great results in the fight against cancer. At present, the use of viral vectors in gene therapy is controversial. Although viral vectors are efficient and easy to integrate into host cells, the infectivity and parasitism of viruses also need our attention [56].

**Figure 7:**
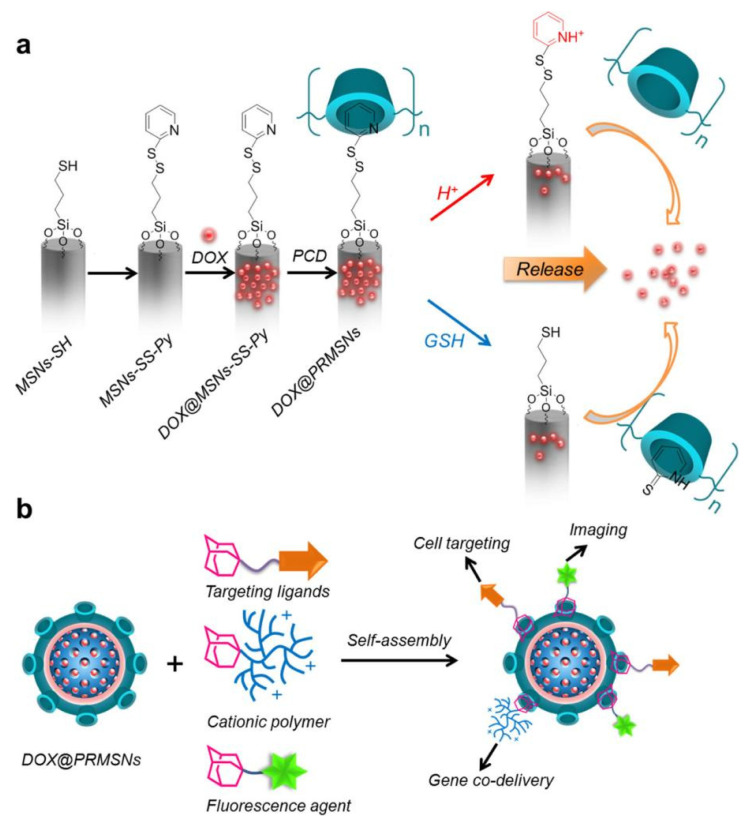
(**a**) PH and redox dual-responsive MSNs’ synthetic procedure, DOX loading, and release mechanisms (DOX@PRMSNs); (**b**) Ad-terminated Guests (targeting ligands, cationic polymer, and fluorescence agent): postfunctionalization of DOX@PRMSNs by self-assembly (reproduced with permission of American Chemical Society from ref. [57]).

In recent years, supramolecular chemistry has been introduced into gene therapy and is thought to be a promising new gene carrier due to its good buffering capacity and biocompatibility. Drug–gene combination therapy strategy has been widely used in the intensive treatment of tumors because of its potential synergistic effect. For example, Liu and colleagues presented a modular approach and designed DOX@PRMSNs via module self-assembly by three different types of adamantane, which rapidly released DOX in lysosomal pH/redox microenvironment and had a significant killing effect on cancer cells. It not only obtained the capacities of gene co-delivery, optical tracking, and cell targeting, but paved the way for individualized and accurate cancer nanomedicine therapies (Figure 7) [57]. A novel injectable supramolecular hydrogel, mPECT(D)/GDDC-4(R)/α-CDgel, combining drug and gene co-delivery had been developed to treat tumors by Peng and co-workers, which was prepared by nanoparticles loaded DOX (mPECT(D)) and GDDC-4/siRNA complexes NPs, based on host–guest interactions between mPEG and α-cyclodextrin. The mPECT(D)/GDDC-4(R)/α-CDgel had shown significant antitumor effects in vivo and in vitro. Confocal laser scanning microscopy verified the release of siRNA and intracellular breakdown of supramolecular polymers, which indicated that the supramolecular hydrogel structure enabled the sustained release of medications and genes, which provided the possibility for co-delivery of them to enhance the effectiveness of cancer therapy [58].

**Figure 8:**
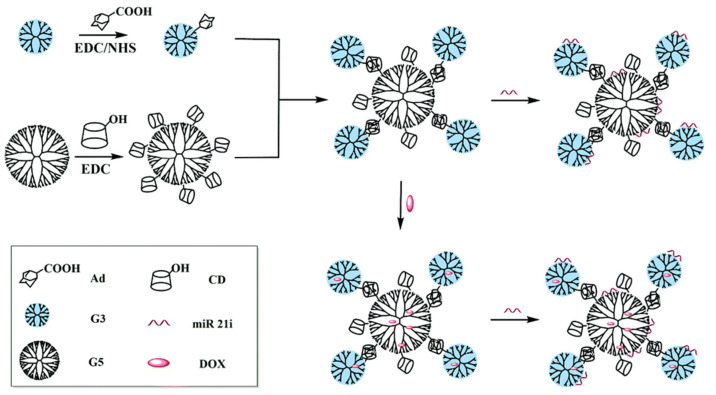
For use in drug delivery applications, G5-CD/G3-Ad CSTDs are synthesized to co-load DOX and miR 21i or to compress miR 21i (reproduced with permission of Royal Society of Chemistry from ref. [59]).

Song and co-workers loaded therapeutic miR21i gene and anti-cancer drug DOX into core-shell tecto dendrimers (CSTDs) formed by supramolecular assembly to achieve co-delivery. CSTDs with each fifth-generation dendrimer surrounded with 4.2 third-generation dendrimers were evaluated as a gene vector, because of the substantial internal volume and superior gene compression capability of CSTDs, the anti-cancer drug DOX could be loaded into its internal cavity, and the electrostatic interaction compressed miR21i. Under the appropriate N/P ratio, CSTDs effectively transfected pDNA and miR21i into cancer cells, and the efficiency of CSTDs/miR21i transfection was at its maximum when the N/P ratio was controlled at 10, which effectively regulated target genes and proteins. The effective transfection of the above genes prevented cancer cells from migrating and triggered their apoptosis. Compared to when DOX was the only substance loaded, the therapeutic impact of cancer cells was dramatically improved via the co-transfection of miR21i and DOX with CSTDs (Figure 8) [59].

Mousazadeh and co-workers found that redox-sensitive folate-appended-polyethylenimine-β-cyclodextrin (roFPC) host–guest supramolecular nanoparticles (HGSNPs) can be created as a tailored co-delivery system of doxorubicin and human telomerase reverse transcriptase-small interfering RNA (hTERT siRNA) for possible cancer therapies. Amantadine-conjugated doxorubicin (Ad-DOX) was loaded into roFPC through supramolecular assembly, and then electrostatically drove self-assembly between hTERT siRNA and roFPC/AdDox to prepare the nanotherapeutic system. The host-guest structure of roFPC allowed for sustained release of pH-dependent intracellular drugs and transfected gene simultaneously, as the embedded siRNA could be released through cleavage of the redox-triggered disulfide bond. The co-delivery vector showed enhanced anti-tumor activity, good water solubility, and biocompatibility of gene transfection combined with DOX. Notably, low N/P levels could be used to target cells that express the folate receptor and promote effective cell apoptosis, which was used in cancer treatments [60].

Similarly, Jiang and co-workers designed ATP-responsive low-molecular-weight polyethylenimine (LMW-PEI) based on host–guest interaction. In the presence of ATP in the target cell, the supramolecular changed from hydrophobic to hydrophilic, allowing the drug to dissociate from the hydrophobic cavity of α-CD while releasing siRNA efficiently [61]. Compared to polyethylene imine, Liang and co-workers developed dual response core/shell nanoparticles based on host–guest interaction, which was more biocompatible. Using HPAA-peptide-Fc as the core and CD-HPG as a shell, they synthesized a cationic carrier by self-assembly, and determined the host–guest interaction by isothermal titration microcalorimetry (ITC). Due to the shielding effect of CD-HPG, HPAA-peptide-HPG demonstrated improved biocompatibility in tests for hemolysis and cell survival, which was favorable for lengthening the cycle of complex in vivo. This effective and secure gene delivery system (HPAA-peptide-HPG) was an excellent illustration of supramolecular used to stimulate responsive siRNA transport [62].

Future clinical developments may benefit from co-delivery of gene and medication by supramolecular nanocarriers as a key technique for improving anti-cancer therapy. While the problem of missing targets has the difficulty of gene therapy, the exploration and study of missing targets of cancer has always been the direction of the medical field and scientific researchers. Wan and co-workers designed a highly efficient supramolecular polymer (SP) to deliver Cas9 ribonucleoprotein. The supramolecular polymer consisted of a host segment formed by β-cyclodextrin-coupled low molecular weight polyethylene imine (PEI), named CD-PEI (CP), and a guest segment formed by a biguidine group and a diamondoid linked by a disulfide bond, named AD-SS-GD. CP/AD-SS-Gd could be obtained by supramolecular coordination reactions. In particular, the presence of multiple strong hydrogen bonds and salt bridges on the surface of this supramolecular polymer enabled CP/AD-SS-GD to interact with Cas9 RNP to create a stable nanocomposite CP/AD-SS-GD/RNP, which was easily released due to the cleavage of disulfide bonds in the intracellular reduction environment, thus guaranteeing effective Cas9 RNP intracellular delivery and release, which efficiently impeded the growth and metastasis of tumors and lessened systemic cytotoxicity [63].

### 2.5. Supramolecular Chemotherapy Combined with Radiation Therapy Based on Cyclodextrins

Radiation therapy is one of the most crucial treatment approaches for tumor treatment, and the main goal is to provide sufficient irradiation to the target area as much as possible and protect the organs at risk to the greatest extent. Therefore, how to transfer the correct dose to the correct position for radiation has always been the development direction and research focus of radiation therapy technology [64]. Scientists had developed a nanotechnology-based pre-targeting strategy to achieve pre-intervention diagnosis and drug targeting through the interaction between the host–guest structures of supramolecular chemistry, which provided the possibility of efficient and accurate local treatment.

Silvia and co-workers took adamantane as the guest, β-cyclodextrin as the host, and adamantane-functionalized macro albumin aggregates (MAA-Ad), radiolabeled Cy5, and β-cyclodextrin (host)-containing PIBMA polymers (^99m^Tc-Cy5_0.5_CD_10_PIBMA_39_) as reaction pairs. After using (^99m^Tc)-MAA-Ad to have a liver or lung embolism, ^99m^Tc-Cy5_0.5_CD_10_PIBMA_39_ was administered intravenously. In vivo experiments demonstrated that the development of MAA-Ad particle accumulation in the lungs or liver resulted in approximately 10-fold accumulation of ^99m^Tc-Cy5_0.5_CD_10_PIBMA_39,_ which indicated that it was possible to direct the degree and direction of ^99m^Tc-Cy5_0.5_CD_10_PIBMA_39_ accumulation by MAA-Ad pre-administration. Due to the formation of supramolecular host–guest interaction between adamantane and cyclodextrin, the preadministration of adamantane guest guided the distribution of cyclodextrin in the host, which improved the accuracy and efficiency of radioembolization in clinical application [65].

### 2.6. Supramolecular Chemotherapy Combined with Protein Therapy Based on Cyclodextrins

Host–guest chemistry has been shown to efficiently introduce the surfaces of mammalian cancer cells, macrophages, and cardiac stem cells in vitro to functionalize living cells. Additionally, host–guest chemistry develops a chemical mechanism for regulating how live cells interact with their surroundings. Since most anti-cancer drugs act in tumor cells, how to achieve intracellular targeted drug release has become an important factor. The following phase in the route of supramolecular functionalization is to deliver chemicals into living cells, which can provide new potential and more effective vaccination administration and immune-modulating drugs.

Li and co-workers prepared a host–guest interaction between γ-cyclodextrin (γ -CD) and coumarin, a new water-soluble, which was supramolecular dendronized polymer (SDP) responding to light. The results of SDP were lysosome-targetable and had excellent biocompatibility in MCF-7 cells. Upon different-wavelength light irradiation, this supramolecular polymer converted between non-covalent and covalent polymers due to coumarin cycloaddition and cleavage reactions. It indicated that the potential uses for the photo-stimulation response material included organelle-targeting applications [66].

Mick M. Welling and co-workers inoculated Ad-functionalized bacteria into muscle or liver through the staphylococcus aureus inoculation model, and these bacteria were targeted intravenously. It was found that the inclusion of Ad-functionalized bacteria (guest) facilitated the accumulation of injected chemicals (host) in both muscle and liver models. This study demonstrated that supramolecular host–guest complexation was used to achieve in vivo substance delivery strategies, and the development and refinement of such strategies provided the conditions for the development of innovative drug delivery concepts through cellular functionalization techniques [67].

What is more, in biological therapy, protein drugs can also be delivered through supramolecular chemistry. In some diseased tissues and organs with active body fluid exchange and catabolism, the harsh environment will quickly eliminate and degrade drugs transported to the site, especially small molecules and protein drugs. Zhu and co-workers developed a multifunctional and bioadhesive polycaprolactone-β-cyclodextrin (PCL-CD), polymersome, which was used to achieve small molecule and protein drug co-delivery. This PCL-CD polymer provided polyvalent cross-linking through surface CD-mediated host–guest interaction to create a supramolecular hydrogel with excellent self-healing and observable shear thinning. In addition, PCL-CD polymers were grafted into biological tissues in situ through surface CD and local guest groups interacting as hosts and guests in the tissue matrix, and thus effectively prolonged the retention time of the materials in grafted tissues. It has been proved that the co-delivery of small molecules and protein drugs through PCL-CD polymers can prevent animal osteoarthritic (OA) knee joints’ cartilage from deteriorating in harsh biochemical conditions and fluid environments [68].

However, it is not enough to deliver protein-based drugs. We hope to release proteins specifically at the site of disease at a given time. Satoshi and co-workers developed an ultrasonic responsive material to regulate the release of a variety of proteins. Two different types of model proteins could be contained by the supramolecular polymeric hydrogel which was crosslinked with a host–guest interaction of β-cyclodextrin and adamantane, and released in a site-specific, stepwise way under the guidance of ultrasound without losing any of their functions. This study provided the possibility for the application of supramolecular hydrogels in protein delivery systems [69].

In addition, supramolecular chemistry can also be involved in biological tissue replacement therapy. Although biomechanically prepared supramolecular polymers have good biocompatibility and biological activity, they are often severely limited by the weak mechanics and brittleness in practical applications. Therefore, many scientists were committed to developing stretchable and tough supramolecular polymers based on cyclodtrins. Monoacrylated β-cyclodextrins with a thiourea (TU) linkage between β-cyclodextrins and acrylates were used as host monomers. Multivalent thiourea-containing host–guest macro-cross-linkers (Tu-HGMCS) can be formed by the self-assembly of such guest polymers and host monomers. Wei and co-workers prepared supramolecular polymers with good mechanical properties cross-linked by TU-HGMCs. The TU-HGMC hydrogel sustained high tensile forces and puncture and quickly restored its original mechanical properties so that it adhered to cells and soft tissues. It was considered to be promising soft tissue substitutes and tissue adhesives, and was widely used in the creation of sophisticated soft biomaterials for tissue restoration, wound dressings, and injury sealing [70].

## 3. Supramolecular Combined Therapy Based on Pillararenes

After cyclodextrin, a novel macrocyclic host molecule, pillared aromatic hydrocarbon, has a highly symmetrical columnar rigid molecular structure and unique host–guest recognition and can be functionalized by a variety of simple and efficient methods. Currently, it has become one of the essential supramolecular building blocks.

As an introductory class of artificial hosts, water-soluble pillararenes [6] (WP[6]A) have been titled “fascinating cyclic aromatic hydrocarbons with a bright future”. The unique columnar molecular structure and electron-rich hydrophobic cavity of pillared aromatic hydrocarbon make it a host-guest complex with various positively charged or neutral guest molecules. However, the terminal groups with easy modification at both ends further broaden the object range and appropriate solvent environment of the column aromatics. Moreover, in converting from the solution phase to the material surface, the diversity of terminal groups dramatically improved the possibility of modification of pillared aromatics on different material surfaces. Hence, multiple responsive supramolecular systems constructed by pillararenes have been continuously designed, synthesized, and reported.

### 3.1. Supramolecular Chemotherapy Based on Pillararenes

Supramolecular chemotherapy is intimately connected with host–guest molecular recognition and uses a supramolecular host combined with a drug–guest to construct anti-cancer drugs. This treatment provides a new idea for cancer treatment with its low toxicity and precise targeting of cancer cells. Interestingly, some markers overexpressed in the intra-tumor environment will bind competitively with the host, and the anti-cancer drug can be released controllably within the cancer cells to restore anti-cancer activity. The depletion of tumor markers further enhances anti-cancer efficiency.

**Figure 9:**
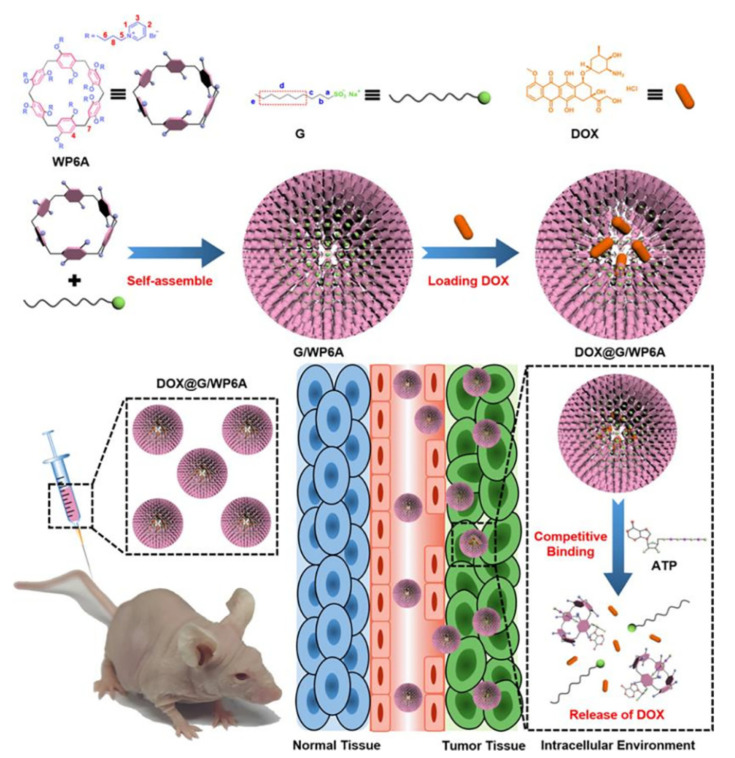
The preparation of supramolecular self−assembled complexes, DDS (DOX@G/WP6A) (reproduced with permission of American Chemical Society from ref. [72]).

For instance, He and co-workers established numerous pillararene-based polyrotaxane [5] to increase the ability of PCL-based supramolecular amphiphiles to load drugs. The supramolecular pseudoblock polymer PR-PAA was prepared using host–visitor collaboration between pillararene-based nonionic polyrotaxane (PR) [5] and β-cyclodextrin end-capped pH-stimulated poly(acrylic acid) (CD-PAA). In an aqueous solution, this supramolecular pseudoblock polymer self-assembled to build PR-PAA-based supramolecular vesicular nanoparticles (PR-SVNPs) and dramatically elevated the drug transportation capacity (DLC, 45.6%) of DOX. The loaded medications might be selectively released in a way that responds to an acidic microenvironment. According to the experimental findings, SMMC-7721’s cytotoxicity and absorption by cells were both increased by PR SVNPs that had been packed with DOX (DOX@PR-SVNPs). More crucially, by taking advantage of numerous desirable properties such as DOX@PR-SVNPs’ strong in vivo anti-tumor activity, extremely low toxicity, and highly effective intratumoral accumulation, supramolecular nanotechnology was applied to drug delivery in scientific research [71]. Chen and co-workers demonstrated the research experience in the preparation of supramolecular self-assembled complexes, DDS (DOX@G/WP6A), which used the host–guest interaction between a cationic aquo-solvable pillararene (WP6A) [6] and a sodium decanesulfonate to encapsulate DOC and form vesicles for medication delivery. Adenosine triphosphate (ATP) competed with WP6A to cause the breakdown of self-assembled vesicles, thereby leading to the subsequent release of DOX in the tumor cells. The advantages of this study were the dynamic noncovalent bonding that conferred brilliant qualities, such as the process’s simplicity and the ease of preparation. Remarkably, the existence of pillararene [6] combined the advantages of host–guest chemistry and self-assembly. Additionally, drug release was stimulated by ATP. For the sake of the environmental differences between tumor cells and normal tissues and EPR effects, targeted drug transport and targeted release within tumor cells were perfectly achieved. Therefore, this supramolecular drug strategy improved the chemotherapeutic result of DOX and reduced the adverse effects, which tells us an illuminating method of cancer treatment (Figure 9) [72].

Using supramolecular self-assembly, Fu and co-workers prepared another class of diselenium-containing ultrathin polymer nanocapsules in light of sidelong crosslinked pillararenes. Depending on the noncovalent connection, supramolecular self-congregations were produced under moderately gentle circumstances, which were reasonable for protein capture. In this study, the new polymer nanocapsules were shown to gain potential anti-cancer effects, good redox reactivity, and biocompatibility with normal cells. On the other hand, the outer layer of the container can be handily adjusted with useful growth entering focusing on ligands through the host–visitor science of pillararenes. The new splendid framework can likewise be created as an insightful and proficient transporter for the designated conveyance of against malignant growth medicates and enriched with additional adaptable restorative properties. To sum up, the preparation of diselenium-containing ultrathin polymer nanocapsules not only furnished a novel anti-cancer drug vehicle for joint anti-cancer therapy but also provided an original tactic for constructing innovative biomaterials [73].

In addition to its role as a carrier of anti-cancer drugs, WPA can regulate biochemical activities and inhibit the proliferation of cancer cells by host–guest interaction, thus achieving the anti-cancer effect. For instance, Li and co-workers initially reported an experimental approach using cationic pillararene [6] (cPA6) that bound to artificial substrates of tyrosine (Tyr) via host–guest interaction, thereby inhibiting Tyr protein phosphorylation and recognizing biochemical regulatory activity. The strong interactions between the cPA6 and pEY were indicated by isothermal titration calorimetry (ITC). It is a concern that the restraint of tyrosine phosphorylation by cPA6 depended on the supramolecular restricting of macrocyclic particles to enzyme substrates. The cPA6-loaded dark phosphorus nanosheet nanocomposites could enter HepG2 cells, actuate apoptosis, and restrain cell expansion by diminishing the degree of Tyr phosphorylation. In addition, the more vital ability of BPNS@cPA6 to inhibit cell proliferation than normal cells exhibited in tumor cells provided a novel idea for anti-cancer therapy. On the other hand, these findings laid the foundation for using columnar aromatic hydrocarbons or their complex to regulate biochemical activities via substrate recognition [74].

Nevertheless, there are many limitations to the traditional nano-drug delivery system and chemotherapy. In particular, single usage of anti-cancer drugs and low drug loading leads to inadequate absorption and significant adverse effects on healthy tissues. To address the issue and to produce creative therapeutic regimens for precision tumor therapy, people committed themselves to developing an intelligent drug delivery system and multiple medication sharing.

**Figure 10:**
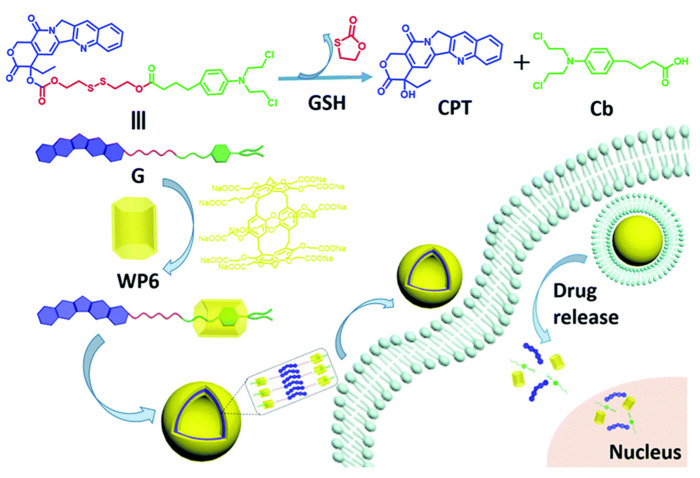
A supramolecular vesicle determined by water-soluble pillararene [6] (WP6) and drug precursors (reproduced with permission of Royal Society of Chemistry from ref. [77]).

Chen and co-workers introduced a new pH-responsive co-encapsulation medication delivery platform to improve drug delivery efficiency in supramolecular combination chemotherapy. A supramolecular amphiphilic compound created from columnar aromatics and an OX-based Pt(IV) prodrug were generated as reactants of (DOX@PtC103CP6A) to build vesicles that successfully encapsulate DOX. Notably, the system passively targeted tumor tissue through enhanced permeability and retention (EPR) effects and released both drugs in an acidic environment to exert synergistic anti-tumor therapeutic effects. Odile Diou and co-workers reported the PLGA-PEG nanocapsules containing a liquid core of perfluorooctyl bromide, which had a similar mechanism [75]. This work highlighted the promising future of supramolecular self-assembled amphiphiles as DDS for combined chemotherapy, providing new ideas for our research [76]. Shao and co-workers reported a supramolecular vesicle determined by aquo-solvable pillararene [6] (WP6) and drug precursors that directly linked two anti-cancer drugs, camptothecin and chlorambucil, via disulfides. In a microenvironment of the tumor where glutathione was overexpressed, disulfide bond breakage resulted in the simultaneous release of both drugs. On the other hand, cytotoxicity tests revealed that these drug–drug binding nanocarriers are superior to individual anti-cancer medications. Furthermore, they dramatically decreased the poisonousness of anti-cancer medications to normal cells in addition to efficiently killing cancer cells. Hydrophobic anti-cancer medicines were given better solubility and bioavailability by using water-soluble macrocyclic host molecules (Figure 10) [77].

### 3.2. Supramolecular Chemotherapy Combined with Chemodynamic Therapy Based on Pillararenes

Chemodynamic therapy is a brand-new group of oncology treatment methods that are rooted in the chemical transformation reaction of tumors’ endogenous chemical byproducts. It uses the tumor microenvironment to activate the Fenton reaction, which generates oxidative hydroxyl radicals for tumor-specific therapy. Recently, the practical idea that the Fenton reaction killed cancer cells by the shift from hydrogen peroxide (H_2_O_2_) to hydroxyl radicals (-OH) within the tumor or cells, thereby increasing intracellular oxidative stress, was widely used in the implementation of chemodynamic therapy (CDT).

**Figure 11:**
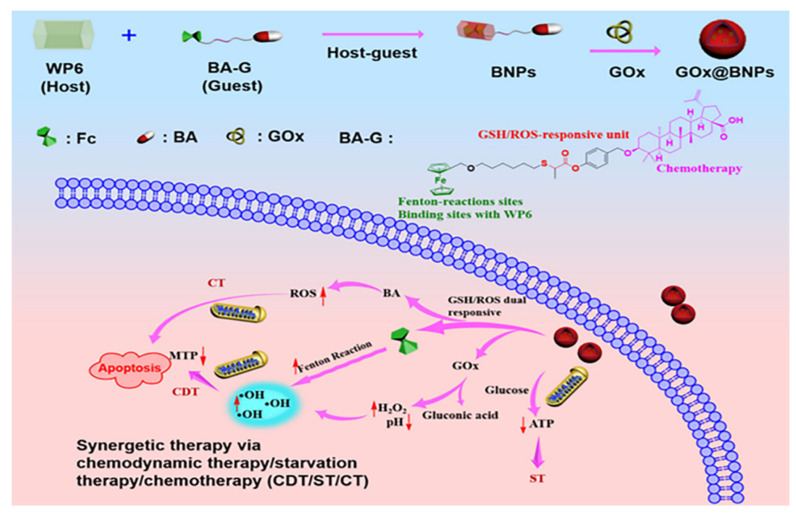
The assembly GSH/ROS dual-responsive supramolecular nanoparticles (GOx@BNPs) (reproduced with permission of MDPI AG from ref. [78]).

For instance, Zhu and co-workers’ research aimed to assemble GSH/ROS dual-responsive supramolecular nanoparticles (GOx@BNPs) to improve the efficiency of both the Fenton reaction and the treatment of CDT. In the work, ferrocene-modified natural anti-cancer compound betulinic acid (BA) prodrug and aquo-solvable pillararene [6] were complexed to form GOx@BNPs, which were then filled with the enzyme glucose oxidase (GOx). The overexpressed GSH and ROS in the tumor microenvironment (TME) can activate the new supramolecular nanoparticles, speeding up the dissociation of nanoparticles. GOx can efficiently and selectively catalyze glucose oxidation to gluconate acid and H_2_O_2_, dramatically influencing the continuous production of ·OH in the Fenton reaction. In summary, the essence of this experiment was to kill cancer cells via depleting glucose necessary for tumor cell growth, improving acidity and H_2_O_2_ levels, thereby elevating CDT to create hydroxyl radicals with eminent oxidative capacity. This kind of multimodal collective treatment pattern of chemodynamic, starvation, and chemotherapy contributed to the advancement of CDT (Figure 11) [78].

Moreover, Ding and co-workers researched preparing pH/ROS dual-responsive supramolecular polypeptide prodrug nanomedicine. They created a supramolecular polypeptide prodrug (SPP-DOX/Ce6) encapsulated with chlorin e6 (Ce6) using a charge-reversal amphiphilic pillararene-modified polypeptide [5] (P5-PLL-DMA) and a ROS-sensitive polypeptide prodrug (P-PLL-DOX) via column [5] aromatic-based host–guest interactions. The pH change caused by the acidic environment within the cell led to a systemic response to release GOD, which played an essential role in catalyzing intra-tumor glucose degradation and Fenton reaction generating • OH for chemodynamic therapy (CDT). Furthermore, the diameters of roughly 200 nm and the surface PEG segment were blamed for the frequent adverse effects and significant tumor accumulation. With a synergistic anti-tumor activity, these supramolecular polypeptide nanoprodrugs provided a viable approach to combination therapy [79].

In addition to the application of targeted cancer therapy, pillararene [6] also has excellent research value in other fields. Gao and co-workers introduced an advanced penetration enhancer for cutaneous drug distribution and construction of an original dermal drug delivery system employing supramolecular nanogels constructed through the identification of host–guest molecule. First, host–guest interactions between the group’s pillararene [5] and alkyl chains on the hyperbranched polyglycerol backbone were regarded as cross-linking medicaments to equip the supramolecular polymer nanogel. Then, the nanogels included the anti-inflammatory medication Dexamethasone (Dexa). Supramolecular nanogels boasted the promise of simple processing, recycling, and self-healing due to dynamic and reversible noncovalent interactions. Supramolecular polymer nanogels can boost the permeability of Nile red penetration through the skin by nine times, according to in vitro skin permeation tests. The study showed an alluring prospect in the increase in the efficiency of transdermal drug delivery systems [80].

### 3.3. Supramolecular Chemotherapy Combined with Photothermal Therapy Based on Pillararenes

**Figure 12:**
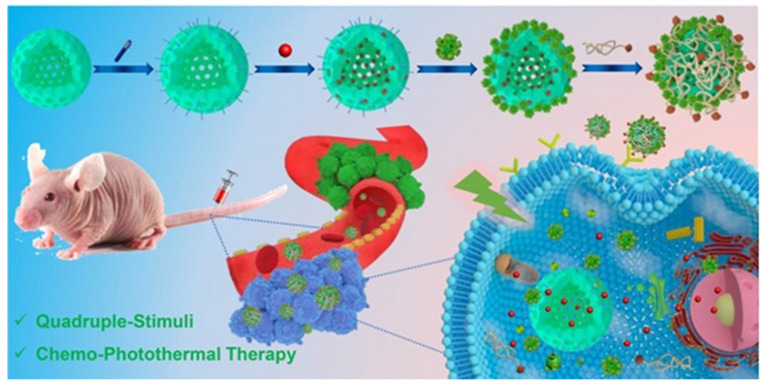
Fa-mPEG@CP5-CuS@HMSN-Py nanoparticles (FaPCH NPs) (reproduced with permission of Ivyspring International Publisher from ref. [82]).

Li and co-workers constructed an intelligent supramolecular nanosystem that combined targeted chemotherapy and photothermal therapy, which were dependent on carboxylato pillararene [5] (CP[5]A)-functionalized CuS nanoparticles (CuS@CP NPs) followed by a further load of chemotherapeutic drug, doxorubicin hydrochloride (DOX). The pH-responsive drug release from CuS@CPG-DOX was facilitated by the decline of DOX-CP[5]A interactions in an acidic environment. This novel nanoparticle showed good photothermal ablation ability for HepG2 cells under 808 nm laser light irradiation. Meanwhile, preliminary in vivo and in vitro experiments indicated that the synergistic use of targeted dual therapies significantly enhanced the therapeutic effect [81]. Fa-mPEG@CP5-CuS@HMSN-Py nanoparticles (FaPCH NPs), a multifunctional supramolecular drug delivery platform described by Yang and co-workers, were constituted by a drug reservoir (HMSN-Py) made of hollow mesoporous silica nanoparticles modified with pyridinium (Py). Subsequently, a layer of near-infrared operable carboxyl column [5] aromatic (CP5) functionalized CuS nanoparticles (CP5-CuS) was attached through a supramolecular host–guest reciprocal action between the CP5 ring and the Py stalk. Furthermore, the introduced folic acid (Fa)-conjugated polyethylene glycol (Fa-PEG) antennas can actively target tumor lesions and achieve synergistic chemotherapeutic therapies characterized by controlled and highly integrated electrostatic interactions. In particular, CP5-CuS acted as a quadruple stimulus-responsive nanochannel for controlled drug release and a dedicated reagent for NIR photothermal therapy. The controllable drug release under quadruple stimuli, including temperature, pH, and competitive binding, was guaranteed by CP5-CuS nanovalves. The multifunctional supramolecular nanoplatform, an emerging cancer therapeutic tool, triggered multimodal synergistic tumor therapy through superior tumor suppression capabilities that were proven effective by in vivo and in vitro results (Figure 12) [82].

### 3.4. Supramolecular Chemotherapy Combined with Photodynamic Therapy Based on Pillararenes

With the help of photosensitizing medications and laser activation, photodynamic treatment (PDT) has been acknowledged as an ongoing method of treating malignant disorders. By activating the photosensitive medications that have been selectively accumulating in the tumor tissue with certain wavelengths of light, the tumor is destroyed by a photochemical process. The new PDT generation of photosensitized medications will impart energy to the ambient oxygen to produce highly active monomorphic oxygen. The surrounding biomolecules and the monomorphic oxygen interact to create cytotoxicity, giving rise to the destruction of the tumor cells. The fact that PDT can deliver accurate and effective treatment with few adverse effects sets it apart from traditional oncology medicine. The unity of chemotherapy and photodynamic therapy ensures efficient and precise treatment. By taking advantage of the biocompatibility of macromolecular nanoparticles, the high permeability of solid tumors, and the upgraded permeability and retention effects of macromolecular nanoparticles, we can deliver the photosensitizer to the tumor site and then administer light to achieve local targeting of the tumor site.

**Figure 13:**
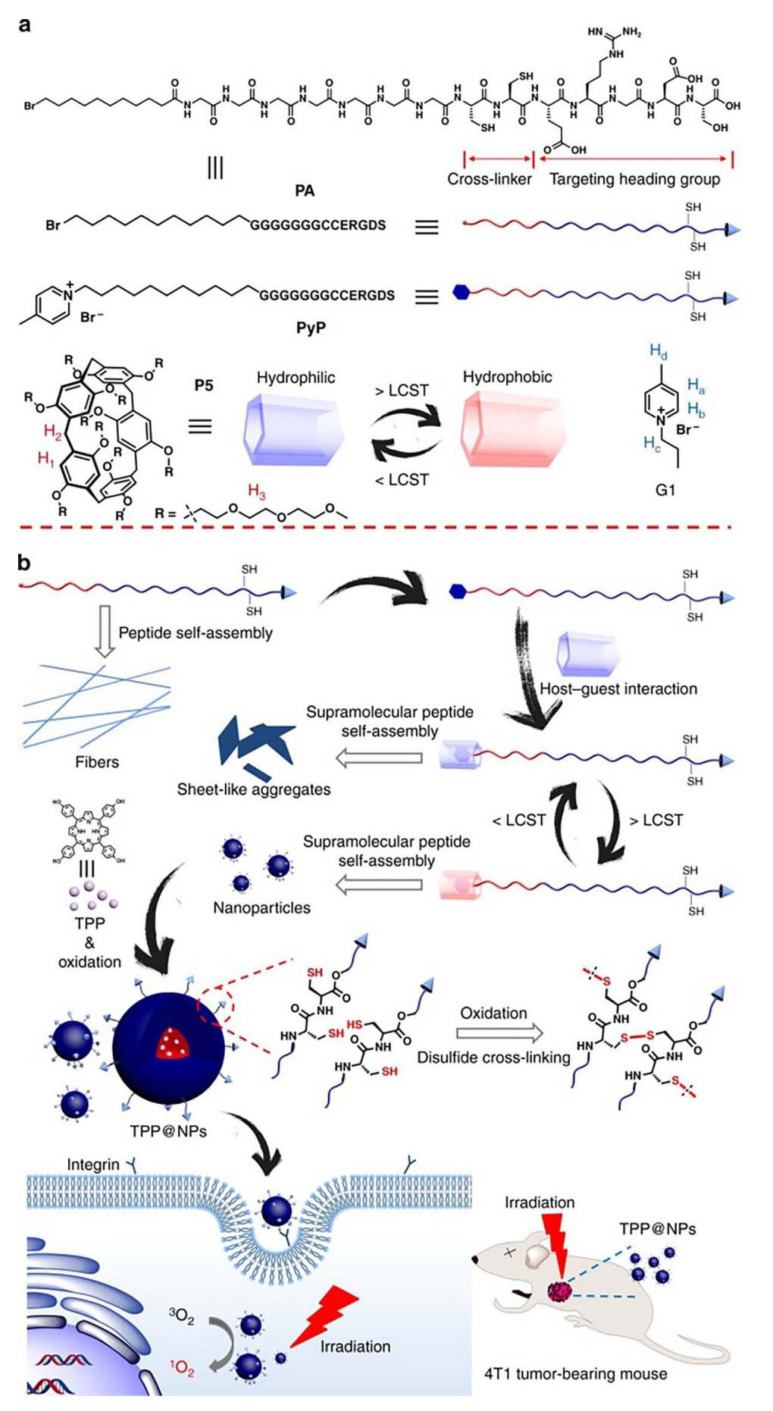
Peptide self-assemblies with multiple nanostructures in photodynamic therapy (**a**) Chemical structures and cartoon representations of PA, PyP, and P5. (**b**) Schematic illustrations of the programmable peptide self-assembly and PDT process. (reproduced with permission of Springer Nature from ref. [83]).

When it comes to supramolecular peptide self-assembly, the current study focuses on how to control the self-assembly morphology and streamline the covalent peptide modification process. Zhu and co-workers created multi-nanostructured peptide self-assemblies in photodynamic therapy. The study involved the construction of a supramolecular peptide using pillararene-based [5] host–guest recognition, which was acknowledged to be an advisable carrier for encapsulating photosensitizers for photodynamic therapy. Self-assembled NPs from supramolecular peptides can be utilized as excellent nanocarriers to stably wrap photosensitizers and improve the effectiveness of photosensitizers for effective tumor ablation. Watchable, pillararene-based supra-amphiphiles assumed the role of a bridge between controlled self-assembly and simplifying the synthesis process through non-commutative interactions. To sum up, this supramolecular peptide is well suited to the requirements of photodynamic therapy because of its simplicity of preparation, lack of purification, stimulus responsiveness, and controllability (Figure 13) [83].

By taking advantage of a pillararene-based [5] supramolecular diblock complex, Wu and co-workers fabricated a dual-stimuli-responsive supramolecular micelle for photodynamic therapy in relevant research. The supramolecular diblock copolymer was designed by the host–guest reciprocity between pillararene [5] and viologen salt, followed by self-assembly and a pack of photosensitizers (pyroph-eophorbide-a, PhA). Consequently, the dual-responsive nanoparticles released PhA in the sour environment at 25 °C, which testified that PhA-loaded nanomicelles presented favorable PDT biopotency and low dark toxicity toward A549 cells. Therefore, this supramolecular diblock copolymer design approach showed great importance and feasibility in improving PDT effectiveness and the construction process for stimulatory drug carriers [84].

### 3.5. Supramolecular Chemotherapy Combined with Radiation Therapy Based on Pillararenes

Radiation therapy (RT) is an ongoing therapeutic method extensively applied to treat malignant tumors and some benign diseases. Furthermore, nanocarriers are materials with nanometer dimensions capable of transporting various drugs and visualizers, which can find application in radiosensitization. Combining the two treatments can effectively improve the efficiency of the fight against cancer.

**Figure 14:**
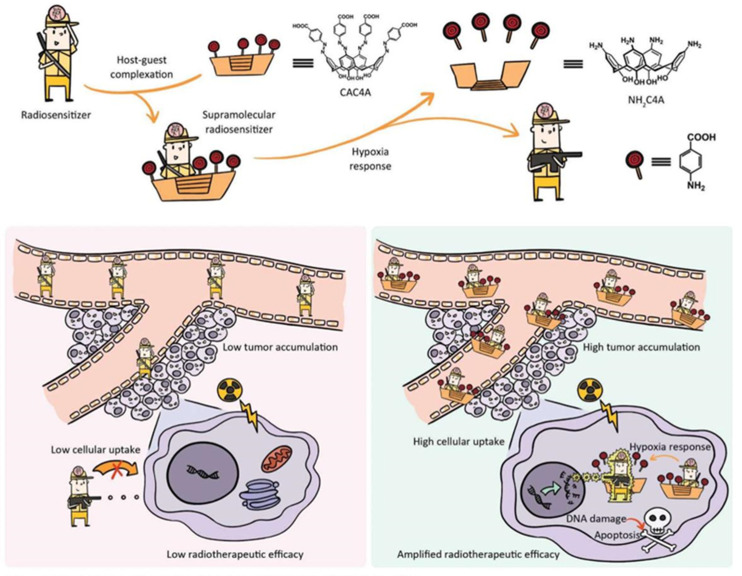
Schematic illustration of the supramolecular radiotherapy strategy based on host–guest chemistry and the corresponding enhanced radio therapeutic efficiency mechanism (reproduced with the permission of John Wiley & Sons, Inc. from ref. [85]).

For instance, to address the problem that the radiation resistance of tumors hinders the effectiveness of radiotherapy, based on the complexation of a hypoxia-responsive macrocycle with a small-molecule radiosensitizer, Hou and co-workers first proposed an original supramolecular radiotherapy approach. The tumor sensitizer banoxantrone dihydrochloride (AQ4N) was produced as a carboxylated azocalixarene [4] (CAC4A) in the study through host–guest interaction. The disjunction of CAC4A-AQ4N led to the release of AQ4N with significant tumor accumulation and effective cellular internalization because the azo group of CAC4A was reduced to the amino group in the tumor’s hypoxic milieu. More importantly, the supramolecular radiation therapy strategy increased the sensitizer enhancement ratio (SER), an essential metric for assessing the sensitizing ability of radiation sensitizers, to an unprecedented 2.349. In the future, the further development of radiosensitizing drugs and supramolecular strategies is of great significance for building an overall system to improve the effectiveness of cancer radiotherapy treatment (Figure 14) [85].

## 4. Supramolecular Combined Therapy Based on Cucurbiturils

**Figure 15:**
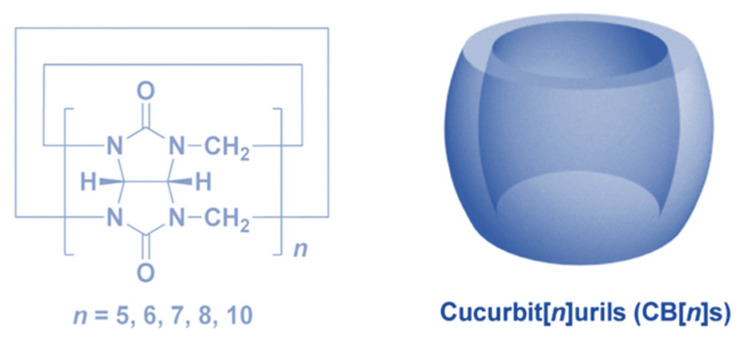
Chemical composition and cartoon representation of cucurbit[n]urils(CB[n]s) (reproduced with permission of Royal Society of Chemistry from ref. [21]).

Cucurbit[n]urils (CB[n]s, *n* = 5–8, 13–15) are macrocyclic capsules created by the combination of glycoluril and formaldehyde which are catalyzed by acid. The characteristic structure of CB[n]s is their extremely symmetric pumpkin-like form, which has a core hydrophobic cavity and a negatively charged carbonyl lining (Figure 15) [21]. It has been reported that CB[n]s can envelope diverse organic and inorganic guest molecules in the hydrophobic cavities of CB[n]s to form 1:1 and 1:2 host–guest complexes [86].

As the result of the collaboration of hydrophobic interactions and ion-dipole interactions, the binding affinities of CB[n]s are substantially stronger than cyclodextrin, whose affinity is dependent on the hydrophobic interaction. Because the polarity of different guest molecules and CB[n]s’ cavity sizes are different, CB[n]s and different guest molecules can form unique host–guest recognitions. CB[n]s have been widely employed to construct drug delivery systems for treating illnesses due to their outstanding molecular recognition and remarkable biocompatibility [87,88,89].

### 4.1. Supramolecular Chemotherapy Based on Cucurbiturils

CB[n]s are mainly utilized as drug carriers within chemotherapy. The complexes formed by CB[n]s and drugs can be competitively dissociated by other guest molecules which have stronger affinities with CB[n]s. Due to the overexpression of spermine in tumor cells, the targeted drug release can be achieved through the high binding force between spermine and CB[n]s.

Yueyue Chen and co-workers have been doing research on CB[n]s for several years. In 2016, a supramolecular method was established by Chen’s group to adjust the cytotoxicity of chemotherapy [90]. In this experiment, dimethyl viologen (MV) was selected as antitumor agent. The loading and release of MV are controlled by the host–guest interaction mediated by the dynamicCB[7]. Without showing any preference, MV is highly cytotoxic to both normal and malignant cells. However, the cytotoxicity of MV to healthy cells can be greatly decreased by enclosing it in the hydrophobic cavity ofCB[7]. The cytotoxic effect of MV could be recovered in the surroundings of tumor cells by incorporating the host–guest composite molecular of MV-CB[7] into tumor cells with overexpressed spermine genes. This finding not only demonstrated that spermine has a great affinity for CB[7], which could lead to the release of MV from MV-CB[7], but it also demonstrated that CB[7] can absorb spermine, which is necessary for tumor cell growth, and further lowers cell viability.

**Figure 16:**
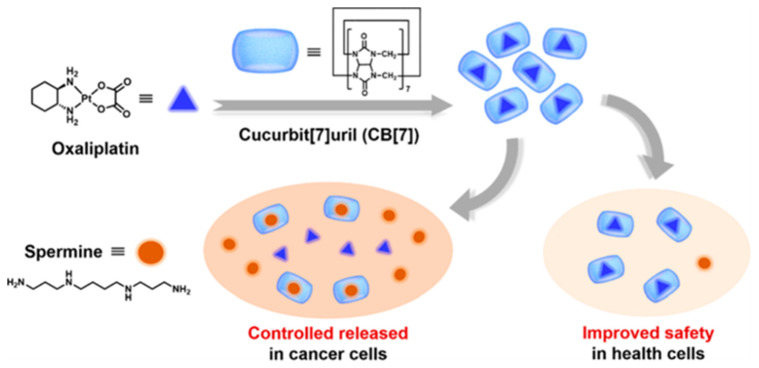
Schematic diagram of oxaliplatin and CB[7] combination and practical application (reproduced with permission of American Chemical Society from ref. [91]).

Based on this research, in 2017, Chen’s group used the clinical drug oxaliplatin as an antitumor agent instead of MV in previous study (Figure 16) [91]. The host–guest complex formation among oxaliplatin and CB[7] was successful in reducing the cytotoxic effects of oxaliplatin towards colorectal healthy cells. Moreover, compound drug molecule oxaliplatin-CB[7] showed stronger antitumor ability than oxaliplatin itself. This phenomenon was consistent with previous research. Accordingly, the overexpressed spermine in colon cancer cells could competitively replace drug molecules in CB[7] to achieve targeted drug release. This strategy was expected to be used with a variety of other therapeutic antitumor medications, providing new opportunities for the prospective use of supramolecular chemotherapy in medical anti-cancer therapy.

**Figure 17:**
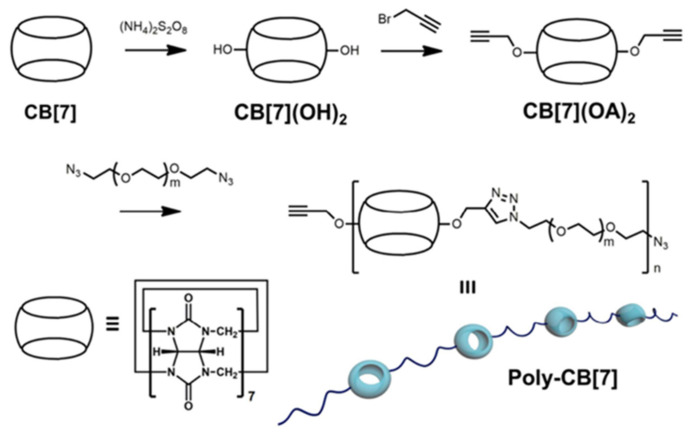
Schematic diagram of synthesizing Poly-CB[7] (reproduced with permission of Elsevier from ref. [92]).

Later in 2017, Chen’s group synthesized a novel class of hydrosoluble high-molecular polymer that contains CB[7] in its principal chain (Figure 17) [92]. By using a copper-catalyzed alkyne-azide cycloaddition click reaction to polymerize bis-alkynyl functionalized CB[7] and α,ω-diazide-PEG, the CB[7]-based principal-chain polymer (poly-CB[7]) was formed. In detail, CB[7](OA)_2_ and α,ω-diazide PEG with a molar ratio of 1:1 were catalyzed by THPTM·CuCl, and the poly-CB[7] was obtained after purification. It was also achievable to encapsulate oxaliplatin inside poly-CB[7]. The properties of releasing drug selectively achieved by host–guest recognition in previous studies are preserved in the supramolecular polymeric complex. Additionally, oxaliplatin can be targeted released in tumor cells which overexpress spermine, while showing low cytotoxicity to normal cells. Similarly, supramolecular polymer complexes are more cytotoxic to cancer cells than oxaliplatin itself. Another advantage of the supramolecular polymer complex compared with oxaliplatin and CB is its long circulation time in vivo. Therefore, this research direction may provide a new idea for supramolecular polymer chemotherapy.

In addition, in 2020, this supramolecular chemotherapeutic drug transport mode also shows good selectivity after replacing the drug with lobacplatin (LbPt) [93]. In this experiment, Chen’s group measured the binding affinity constant (*K*_a_) of CB[7] and LbPt, as well as CB[7]and spermine. Data from isothermal titration calorimetry (ITC) experiments demonstrate that CB[7] and spermine had a *K*_a_ of (1.18 ± 0.12) × 10^6^ M^−1^, which is a factor of five higher than *K*_a_ of CB[7] and LbPt, which was (2.09 ± 0.07) × 10^5^ M^−1^. This result confirmed that CB[7] molecule containing LbPt could spontaneously and competitively bind with spermine to achieve the purpose of targeted drug release. This study shows that LbPt and oxaliplatin can both be combined with supramolecular chemotherapy.

**Figure 18:**
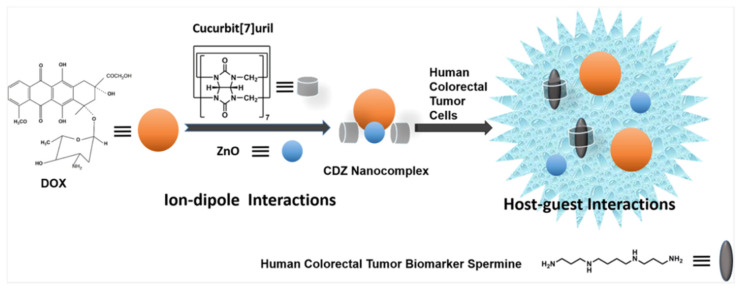
Illustration of DOX, ZnO, and CB[7] connections and drug delivery system (reproduced with permission of American Chemical Society from ref. [95]).

Recently, Chen’s group has extended the utilization of CB[7] to two new kinds of anti-cancer drugs, heptaplatin [94], and doxorubicin (DOX) [95]. In the study using heptaplatin, by ^1^H NMR, heptaplatin revealed a significant affinity to CB[7], with a *K*_a_ of (1.38 ± 0.06) × 10^6^ M^−1^. At pH 6.0, the overexpressed spermine in the tumor microenvironment could competitively swap heptaplatin from heptaplatin-CB[7]. Furthermore, according to research on the anti-cancer processes of supramolecular complexes, heptaplatin-CB[7] induced early apoptosis of human colorectal tumor cells (87.73%) and the inhibition reaction during G1 phase of the malignant cell cycle, which proved heptaplatin-CB[7] is highly cytotoxic against human colorectal tumor cells, but low cytotoxic to normal human colorectal cells. Limited by the pore size of CB hydrophobic cavity, Chen’s team achieved directional release of DOX through non-covalent interaction (Figure 18). In this experiment, DOX was first combined with ZnO to form DOX-ZnO, and then combined with CB[7] through ZnO to form DOX-ZnO-CB[7](CDZ) nanocomposite. On account of the high binding affinity between spermine and CB[7], DOX could be specifically released in tumor cells with spermine overexpression. In this study, the antitumor agents used in previous studies were extended from platinum to non-platinum, and DOX and CB[7] were linked by ZnO, which achieved targeted release of the drug and reduced toxicity to normal cells.

CB[n] can be used for anti-cancer drug delivery mainly due to its high tolerance, high stability, and easy water solubility with anti-cancer drug conjugate. Mantao Chen et al. measured the aqueous solubility and stability of DOX and camptothecin (CPT) before and after mixing with CB, and the results showed that these qualities had significantly improved after supramolecular modification while effectively maintaining the anti-cancer activities of chemotherapy drugs [86]. The observation of nuclear Overhauser factor correlative sensory information between CB[7] and 3-methylcyclohexylamine demonstrated that hostage–guest complex formation took place between CB[7] and 3-methylcyclohexylamine and the signals of guest molecules penetrating deep into the CB[7] were captured. The *K_a_* value of binding CB[7] and 3-methylcyclohexylamine was (2.73 ± 0.84) × 10^6^ M^−1^ by the ITC experiment. It was discovered via research into the shape of supramolecular nanomaterials in water that hydrophilic CB[7] increased the aqueous solubility of DOX. Transmission electron microscopy (TEM) and dynamic light scattering (DLS) data revealed the same conclusion: supramolecular nanomedicine is very stable in physiological environments. In addition to DOX, the host–guest interaction among CPT and CB[7] was confirmed using biological layer interferometry. At last, the therapeutic effect of supramolecular nanomedicine on HeLa and U87 cells was evaluated by 3-(4′,5′dimethylthiazole-2′-yl)-2, 5-diphenyltetrazolium bromide (MTT) experiment. The anti-cancer activity of DOX and CPT was well maintained.

**Figure 19:**
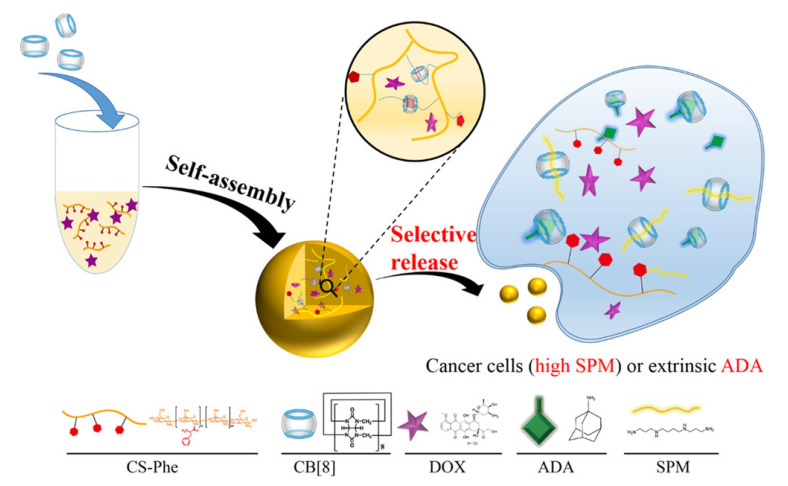
Illustration of preparation of supramolecular CNGs triggered by host–guest interaction and release of stimulated response loads in cancer cells (reproduced with permission of American Chemical Society from ref. [88]).

Besides applying CB[n] as a drug carrier, CB[n] can also be combined with other molecules to achieve supramolecular chemotherapy due to its high stability and host–guest recognition. Ding et al. used CB[8] and chitosan to construct a drug delivery system carrying DOX for directional release [88]. In this project, chitosan (CS-PHE) grafted with two phenylalanine (Phe) units were packaged in a lumen of CB[8] in aqueous solution to promote CS-PHE cross-linking and formation of CNGs. DOX was encapsulated in CNGs matrix in the time of the preparation of nanogels, and the remarkable drug-loading efficiency of DOX-CNGs was obtained. As shown in Figure 19, CS-PHE was first prepared in this experiment. Under slightly acidic conditions, CB[8] was added into the water solution of CS-phe to form chitosan nanogels (CNGs). CB[8] was utilized as a cross-linking agent which would pull two phenylalanine units of CS-phe into its cavity. During preparation, DOX, an anti-cancer chemotherapy drug, was immobilized in the CNGs matrix. The results showed that CNGs and DOX-CNGs had outstanding colloidal stability in water solution, and DOX-CNGs showed the required cytotoxicity when stimulated with SPM or Adenosine deaminase (ADA), indicating that the system had great potential for release under controlled loading.

**Figure 20:**
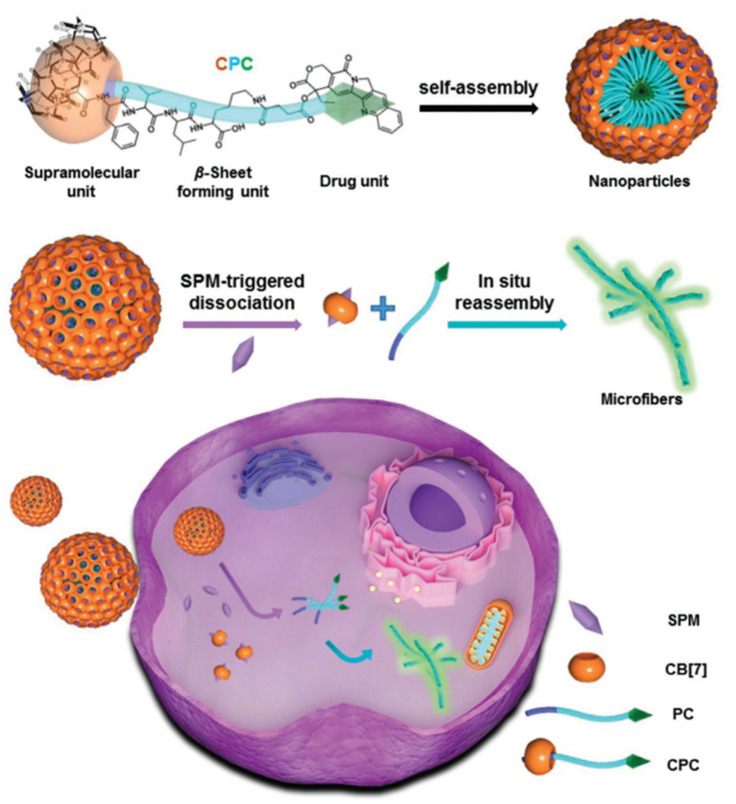
Schematic diagram of CPC drug synthesis and mechanism of function (reproduced with permission of John Wiley & Sons, Inc. from ref. [96]).

For CPT, Sun et al. constructed a new supramolecular peptide-derived nanodrug (SPN) using the non-covalent interaction between CB[7] and a camptothecin conjugate named FFVLK-CPT (PC) [96]. As shown in Figure 20, different from the traditional practice of placing the drug unit in the cavity of CB[7], this project links the drug unit to the FFVLK peptide, and then the CB[7]-FFVLK-CPT complex compound (CPC) is created when the n-terminal residue (Phe) of the FFVLK peptide is encapsulated by CB[7] through host–guest interaction. The complex compound can form nanoparticle anti-cancer drugs through self-polymerization. In cancer cells overexpressing SPM, this drug is based on the principle that SPM can competitively replace the Phe group in CB[7], leading to the dissociation and release of CPT by nano-anti-cancer drugs, and realizing the oriented effect of CPT. Through cytotoxicity assay, the nanomaterial can decrease the systemic toxicity of CPT in vivo, and significantly increase the cumulation and storage of CPT in cancer cells.

**Figure 21:**
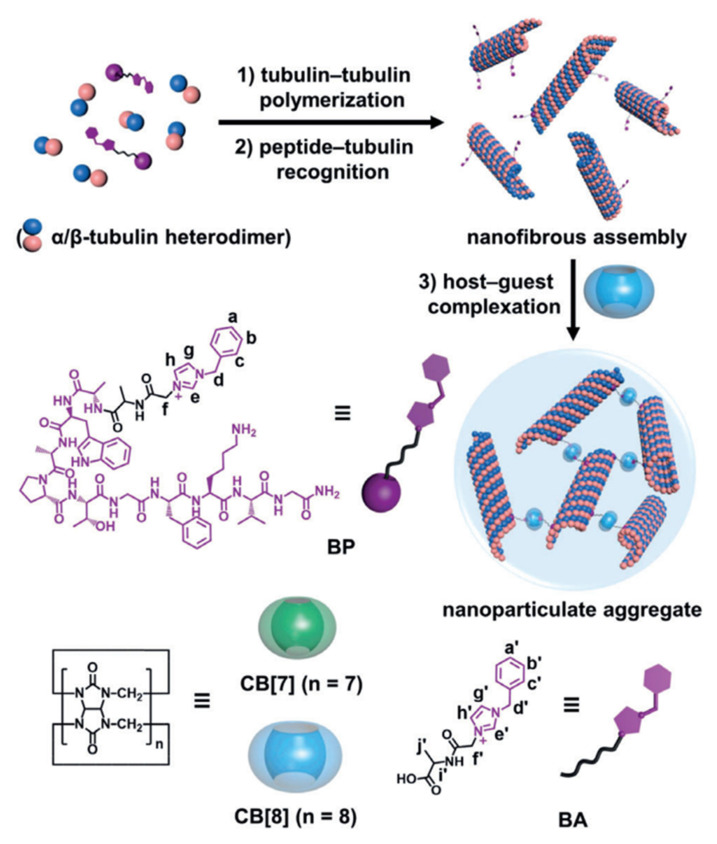
Schematic diagram of ternary supramolecular (BP-CB[8])@MT combination for targeting microtubule assembly (reproduced with permission of John Wiley & Sons, Inc. from ref. [97]).

In addition to the direct control of drug release to accomplish the aim of targeted therapy, Zhang et al. designed a scheme [97]. On the basis of the principle that competitive binding of anti-mitotic polypeptide (BP) with α-tubulin subunit can show anti-mitotic activity, this scheme uses CB[7] and CB[8] to connect chemically modified BP to induce microtubules (MT) to change from linear polymer to spherical nanostructure, which can be used to fight MT aggregation-related diseases in clinical practice. As shown in Figure 21, in this assay, the 1-benzyl imidazole group was first covalently bound to the N-terminus of tubulin-targeted anti-mitotic peptides through additional links containing acyl-methyl and alanine residues. ITC experiment results show that CB[8] has a strong binding affinity with benzimidazole unit. These bioessential fragments with appropriate molecular length can promote the complete 1:2 homologous complexation of CB[8] with the benzyl imidazole fraction of BP peptide while ensuring the specific interaction between the peptide and tubulin. Morphological studies have shown that BP and CB[8] can complexate at a ratio of 2:1 to achieve the intertubule aggregation of MTs. Extensive supramolecular MT cross-linking has been proven to produce considerable apoptosis and eventually suppress tumor growth by in vitro and in vivo experiments.

### 4.2. Supramolecular Chemotherapy Combined with Phototherapy Based on Cucurbiturils

As mentioned above, as a popular therapy in phototherapy, photodynamic therapy (PDT) is an anti-cancer therapeutic method which utilizes photosensitizers to produce a great quantity of reactive oxygen species (ROs) under laser irradiation at a specific wavelength, and participates in autophagic cell death by activating the death receptor pathway and apoptotic mitochondrial pathway. It is noninvasive, has low systemic toxicity, and is highly selective. In a word, PDT typically includes the site-specific treatment using photosensitizers, which, when exposed to light in the presence of molecular oxygen, would produce extremely lethal singlet oxygen (^1^O_2_).

**Figure 22:**
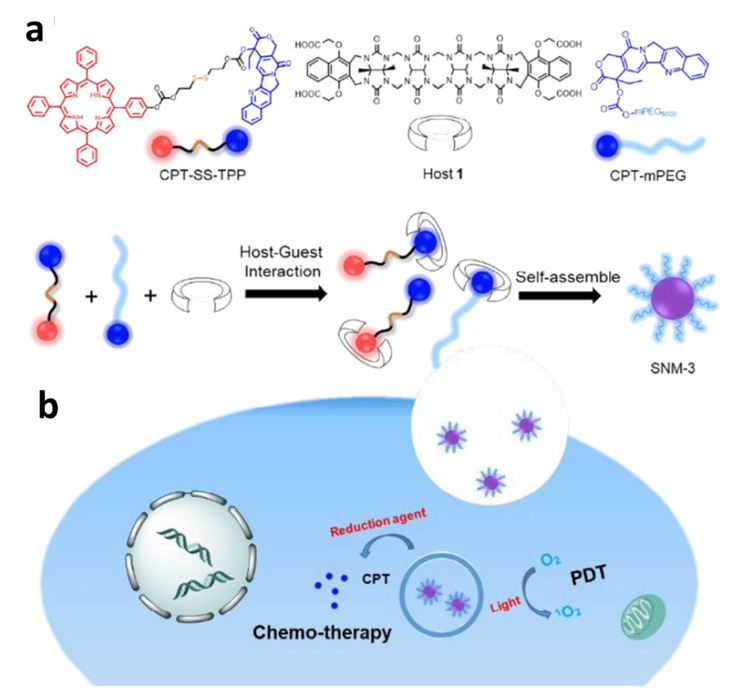
(**a**) Chemical structure and schematic diagram of CPT-SS-TPP, CPT-mPEG, and host 1; (**b**) Schematic diagram of chemophotodynamic combination therapeutic method (reproduced with permission of Royal Society of Chemistry from ref. [98]).

To solve the problem that the highly cytotoxic ^1^O_2_ produced by photosensitizer in PDT would be suppressed by aggregation-caused quenching (ACQ) effect and affect the treatment effect, using the host–guest interaction of CB[n] Mao et al. established a supramolecular nanomaterial for chemical-photodynamic combination therapy [98]. In this protocol, the PDT photosensitizer utilized was tetraphenylporphyrin (TPP), and the chemotherapeutic drug employed was CPT. A redox disulfide linker (CPT-SS-TPP) connected CPT and TPP. CPT-mPEG was obtained by coupling methoxyl polyethylene glycol (ethylene glycol) with CPT. To improve physiological stability, CPT-mPEG was then added to supramolecular nanomaterials. A ternary supramolecular nanomaterial (SNM-3) was created by the supramolecular assembly of CPT-SS-TPP and CPT-mPEG through the mediation of CB[n] host 1. SNM-3 can release CPT and TPP in an intracellular reducing environment (Figure 22), thereby improving the combination therapeutic effect. According to the studies on the host–guest interaction among host 1 and CPT or TPP, the ratio of CB[n] to CPT is 1:1, and host 1 can coat CPT and TPP with a pretty much identical binding force to promote the synthesis of supramolecular nanomaterials. After that, CPT-SS-TPP, CPT-mPEG, and host 1 were used to prepare SNM-3 according to the molar ratio of 2:1:1. In addition, in this experiment, CPT-SS-TPP and CPT-mPEG were used to prepare the supramolecular nanomaterials SNM-2 without the use of host 1. SNM-2 was a disorderly aggregate with different percentages of the two constituent parts, according to TEM images, so host 1 was crucial for the formation of ordered supramolecular nanomedical medicine. Finally, cell culture experiments were performed to evaluate the efficacy of combined treatment. The results showed that compared with CPT or TPP-OH, the combined action of chemical and photodynamic, which was achieved in SNM-3, enhanced the efficacy and reduced the side effects.

**Figure 23:**
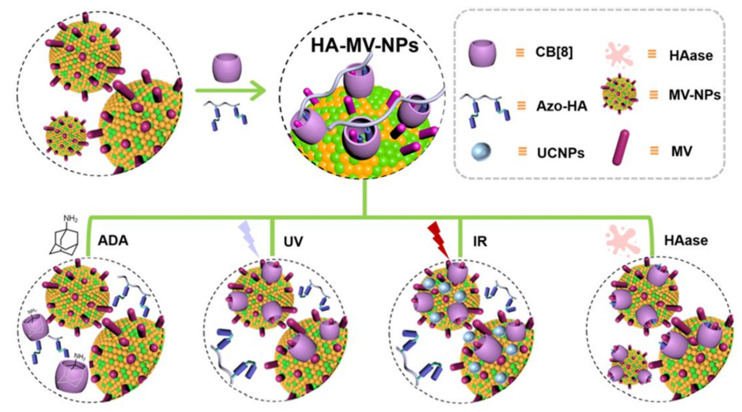
Schematic diagram of preparation and application of HA-MV-NPs (reproduced with permission of American Chemical Society from ref. [99]).

In addition to PDT, traditional photochemotherapy methods have also made progress. Gao et al. established a three-host–guest complex through multi-stimulus response, including hyaluronan (HA)-interlayer MV nanoparticles (HA-MV-NPs), trans-azobenzene conjugated HA(Azo-HA), and polymer NPs (MV-NPs) with polylactic acid on the MV-terminal, which were all combined by CB[8] [99]. In this project, MV-terminal polylactic acid and Azo-HA were primarily prepared, and then MV-NPs were made by the emulsion method. According to the study on the drug model, the molar ratio of MV-NPs, Azo-HA, and CB[8] was 1:1:5 to prepare HA-MV-NPs. MV on the surface of MV-NPs is wrapped by CB[8] and Azo-HA without showing cytotoxicity (Figure 23). Under the conditions of ADA, UV, IR, or Hyaluronidase (HAase) treatment, CB[8] or HA will fall off from the surface of MV-NPs, resulting in cytotoxicity.

This protocol can be applied in agricultural and biomedical fields. Under the action of ultraviolet (or sunlight), HA-MV-NPs can be stimulated by selective decode-induced activation (DIA), showing particular herbicidal activity, and it is a human-harmless weedicide. When used in chemotherapy, HA-MV-NPs have 21 kinds of selective DIA against the hyaluronidase, released particularly by some strains such as staphylococcus aureus in vitro and in vivo, and have a specific ability to resist S. aureus. Additionally, after incorporating upconverted nanoparticles (UCNPs) 22 with HA-MV-NPs, which were able to enter tissues more deeply and translate into UV irradiation in the UCNPs field, selective DIA for cancer in reaction to far-infrared (IR) irradiation was subsequently demonstrated in vivo. This high-molecular polymer laminate NPs showed a response to both local and distant stimuli and is targeted to a variety of medically relevant applications using the DIA approach. This process was mediated by host–guest interaction.

### 4.3. Supramolecular Chemotherapy Combined with Thermal Therapy Based on Cucurbiturils

Thermal therapy is an important part of today’s cancer treatment, which has fewer side effects than chemotherapy and radiation, and can reach deeper, less accessible tumors. The effectiveness of cancer therapy has been demonstrated to be enhanced by using hyperthermia, which involves heating tumor tissue to 41 to 45 °C, and thermoablation, which involves heating the tumor to more than 50 °C to cause cell necrosis [100,101]. Phototherapy can be easily combined with thermal therapy, thus forming a new therapy, namely photothermal therapy mentioned above. In order to achieve the goal of treatment, the basic idea behind photothermal therapy is to concentrate materials with high photothermal conversion efficiency close to the tumor through targeted identification. The photothermal conversion can then be accomplished by applying a specific light irradiation.

**Figure 24:**
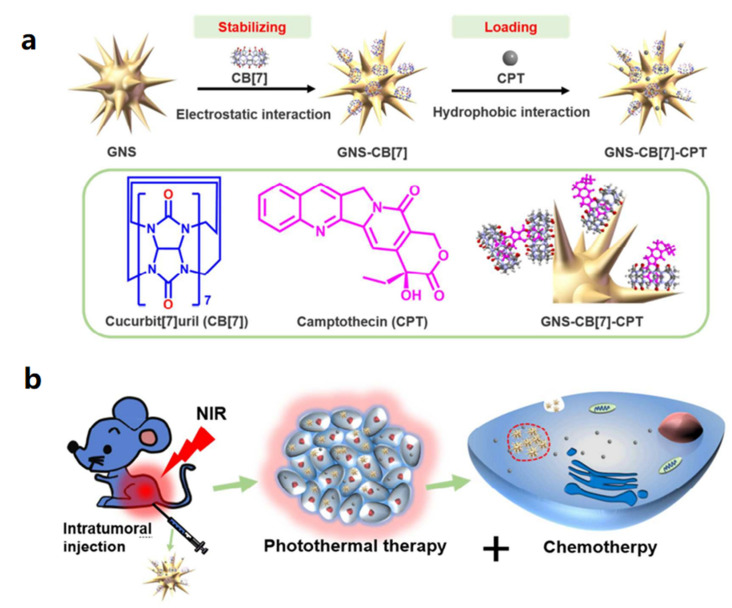
Diagram of (**a**) the synthesis process of GNS-CB[7]-CPT. (**b**) Pharmacokinetics and photothermal treatment (reproduced with permission of American Chemical Society from ref. [102]).

Xu et al. developed a near-infrared (NIR)-triggered approach to regulate medication release from a supramolecular assembly system to achieve synergetic chemotherapy-photothermal treatment of cancer [102]. In this project, a stable gold nanostar (GNS) was designed using CB[7] to encapsulate the anti-cancer medication CPT via host–guest interaction. In addition to improving the stability of GNS in aqueous solution, CB[7] can act as a cage for the intermolecular assembly of CPT. Furthermore, NIR irradiation can be used to increase the release of the medication in the absence of competitive complexation. The photothermal impact of GNS in combination with chemotherapy can effectively cure malignancies. As shown in Figure 24, CB[7] was first stabilized on the surface of GNS through electron-rich carbonyl channels to form GNS-CB[8], and then GNS-CB[7]-CPT was created by encapsulating CPT by hydrophobic contact within the cavity of CB[7]. Sodium citrate was used to reduce chloroauric acid to create gold nanoparticles. Gold nanoparticle seeds, silver nitrate, ascorbic acid, and hydrochloric acid were all added one at a time to create GNS with a star anisotropic structure. Then, to enclose CPT molecules, CB[7] was placed onto the exposed GNS surface. GNS may be employed to accomplish the goal of photothermal conversion due to its many crisp branches, high photothermal conversion, strong LSPR, and near infrared absorption. CPT release may be triggered by near-infrared (NIR) light passing through GNS. Additionally, cancer can be effectively treated by combining NIR light irradiation of the GNS with the hyperthermia brought on by CPT chemotherapy.

**Figure 25:**
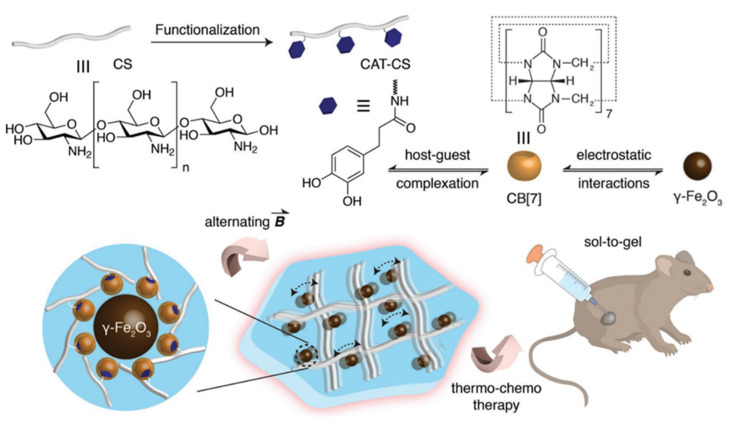
Diagram of magnetic hydrogel nanocomposites made by molecular recognition and electrostatic interaction with CB[7] (reproduced with permission of John Wiley & Sons, Inc., from ref. [101]).

In addition to the photothermal transformation applying NIR, Qiao et al. used CB[7] and catechol functionalized chitosan to realize the functions of hyperthermia and chemotherapy under the action of alternating magnetic field through the role of host and guest recognition [101]. The scheme is the superparamagnetic γ-Fe_2_O_3_ nanoparticles “stick” on the catechol-functionalized chitosan polymerization backbone. In this network, CB[7] not only promotes the host and guest recognition of catecol derivatives but also generates a potent supramolecular electrical interaction between its carbonyl entrance and γ-Fe_2_O_3_ nanoparticles, maintaining the nanoparticles’ original physical and chemical characteristics. Under alternating magnetic fields, γ-Fe_2_O_3_ nanoparticles show vibrational motion and heat, resulting in a combined thermal and chemotherapeutic supramolecular hydrogel. As shown in Figure 25, the connection among catechol-functionalized chitosan (CAT-Cs) and superparamagnetic γ-Fe_2_O_3_ nanoparticles was promoted by the use of CB[7] as a non-covalent linker. CAT-CS, a polyvalent side-chain functional polymer containing atechol derivatives, was synthesized by using the postfunctionalization of natural polysaccharide chitosan. After the addition of glycerophosphatate (GP), the polymer formed a thermoresponsive hydrogel CAT functional group, which was able to be feasibly identified and packaged by CB[7]. However, the carbonyl inlet of CB[7] bound with γ-Fe_2_O_3_ nanoparticles through electrostatic interaction, forming a non-covalent bond connection network between polymer skeleton and nanoparticles. The data of experiments showed that compared with the efficient release of Dox demonstrated by hybrid supramolecular hydrogel (HSH), the samples without CB[7] introduced into the non-covalent interaction between nanoparticles and polymeric backchain showed a slower release rate, which demonstrated the importance of CB[7], which could be applied as a non-covalent linker in HSH assembly.

**Figure 26:**
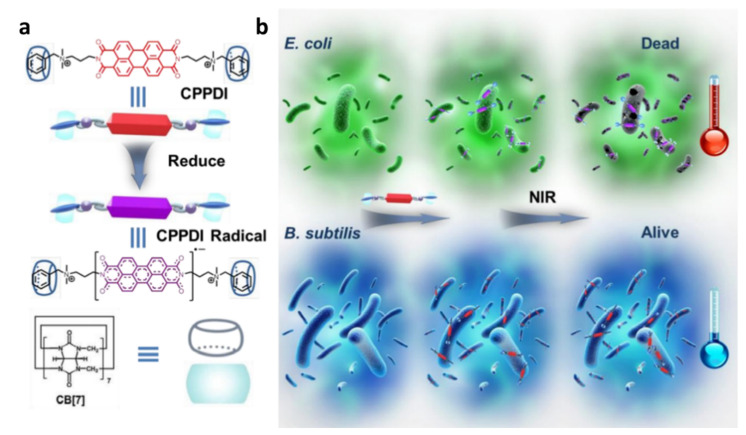
Diagram of (**a**) the chemical structures of CPPDI and CPPDI radical anions; (**b**) photothermal therapy for CPPDI with high selectivity towards *E. coli* over *B. subtilis* (reproduced with permission of John Wiley & Sons, Inc. from ref. [104]).

CB[n] can be applied not only in cancer treatment, but also in reducing antibiotic resistance via photothermal therapy. Photothermal therapy causes the surface of strain to heat up by photothermal materials and denatures its proteins, which leads to the death of strain, and has the advantage of avoiding the development of drug resistance [103]. Yang and co-workers used perylene diimide derivative (PPDI) and CB[7] to construct supramolecular complex (CPPDI) through host–guest interaction (Figure 26) [104]. The experiments confirmed that facultative anaerobic bacteria such as *E. coli* have the reducing ability to trigger CPPDI to generate CPPDI radical anion, which could be used for photothermal therapy under NIR irradiation, causing the death of *E. coli*. In contrast, bacteria such as *B. subtilis* cannot provide sufficient reductive ability to initiate the antimicrobial action of CPPDI, indicating that CPPDI is selective for different bacteria. By using photothermal therapy, CPPDI effectively avoided the problem of resistance associated with using conventional antibiotics.

**Figure 27:**
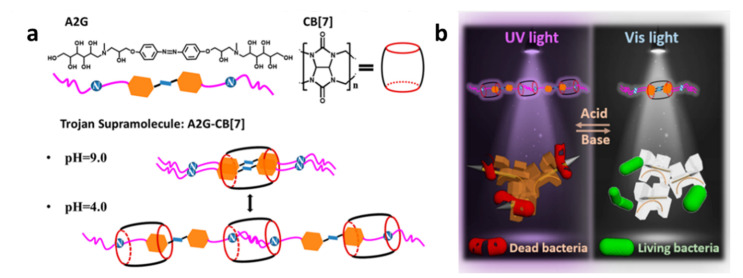
Diagram of (**a**) the chemical structures of A2G, CB[7], and the formation process of A2G-CB[7]; (**b**) effects of A2G-CB[7] on bacteria under different pH and light conditions (reproduced with permission of American Chemical Society from ref. [105]).

By utilizing light irradiation to adjust, Wang and co-workers reported an antibiotic which was capable of producing benign or bactericidal effects on bacteria under different conditions via the host–guest interaction between CB[7] and azobenzene group (Figure 27) [105]. The Azo group could be used as a photoswitch in this reagent, which could be combined with the nitrogen atom of glycoamine head as a pH switch to improve the sterilization efficiency. CB[7]was used to reduce the inherent toxicity of the Azo group to bacteria when the photoswitch and pH switch were not triggered. The composed A2G-CB[7] supramolecule is harmless to bacteria under the weak base condition of pH9.0, but under the irradiation of pH4.0 and 365 nm UV, it exhibited excellent bactericidal ability due to the protonation of glycoamine head and the slip of CB[7] from the Azo group. The bactericidal behavior of supramolecules ceased when UV irradiation was removed, and the pH was raised to a weakly alkaline level. This protocol could control antibacterial action by regulating environmental factors, avoiding the accumulation of antibiotics in the environment, and thus reducing the chance of bacterial mutation fighting against antibiotic resistance.

## 5. Supramolecular Combination Therapy Based on Metal Coordination Complexes

Biomacromolecule drugs have become one of the important medications for the therapeutics of human diseases. The choice of an adequate drug delivery method is essential to ensuring the therapeutic efficacy of biomacromolecule medications in vivo due to the high tendency of biomacromolecule pharmaceuticals to degrade in the human body. At present, the studies on drug-targeted release systems based on metal coordination complex are becoming more and more diverse. These methods include metal-organic frameworks (MOFs), supramolecular coordination complexes (SCCs), and hierarchical self-assembly (HSA) based on SCCs [106,107,108]. These three approaches will be reviewed below.

### 5.1. Supramolecular Combination Therapy Based on Metal-Organic Frameworks

MOF is a rising category of supramolecular host materials, the benefits of which include a facile surface functionalization process, high encapsulation efficiency, regulated drug release, and excellent biocompatibility [106]. Therefore, MOF has been extensively developed in recent years, as has the medication delivery system built upon it.

MOFs are porous materials created by means of the pathway that metal cations or clusters are coordinated with organic ligands [109,110]. Several macromolecular MOF materials have been created successively with clear structure and function because of the advancement of supramolecular host–guest chemistry. By causing biological macromolecules to self-assemble in a mixture of metal ions and organic ligands, these MOF materials can encapsulate biological macromolecules into their interior spaces. Moreover, due to the adaptability of coordination chemistry, these biological macromolecules, as guest molecules, can be successfully integrated into the lumen of host molecules, namely, MOFs, thus producing a host–guest system (Biomacromolecules@MOFs) with stable structure. So far, compared with cyclodextrin, crown ether and calixarene, less research has been conducted upon host–guest systems for biological macromolecules relying on MOFs.

**Figure 28:**
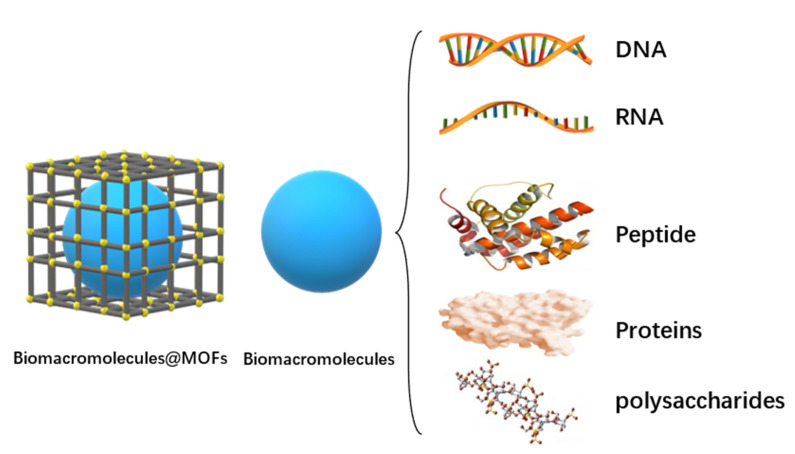
Morphology diagram of biomacromolecules@MOFs and biomacromolecules types.

As shown in Figure 28, MOFs can combine with biomacromolecules to form “cage” structures on the outside and synthesize Biomacromolecules@MOFs molecules. By controlling the release of biomacromolecules in the human body, immunotherapy, starvation therapy, antioxidant therapy, gene therapy, multi-channel collaborative therapy, and other purposes are realized. Biomacromolecules in MOFs cavities can be DNA, RNA, peptides, proteins, polysaccharides, and other biological macromolecules, depending on their efficacy.

There are two ways to synthesize biomacromolecules@MOFs. The first is pore encapsulation, which involves inserting biomacromolecules into the cavity of prefabricated MOFs. This method requires biomacromolecules to be consistent with the 3D shape of the cavity of MOFs. The second is in situ encapsulation. In situ encapsulation, which serves as an alternative to pore encapsulation, can absorb a wider variety of biomacromolecules, regardless of their size. The basic idea is that biomolecules are evenly encased in MOF materials after being self-assembled around biomacromolecules by MOF precursors, and eventually results in host–guest supramolecules.

Using pore encapsulation, Hu et al. synthesized a MOF material, Mg(H_2_TBAPy)(H_2_O)_3_·C_4_H_8_O_2_ (TDL-Mg) [111], and then successfully loaded 5-fluorouracil (5-FU) into TDL-MG using the abundant π binding sites provided by TDL-MG. Multidirectional hydrogen bonds, numerous π-interactions, and the synergy of restricted channels produced appropriate host–guest interactions. Within 72 h, the microthermal technique released 76% of the load, which is a medically acceptable rate. Drug release is achieved by regulating the temperature.

**Figure 29:**
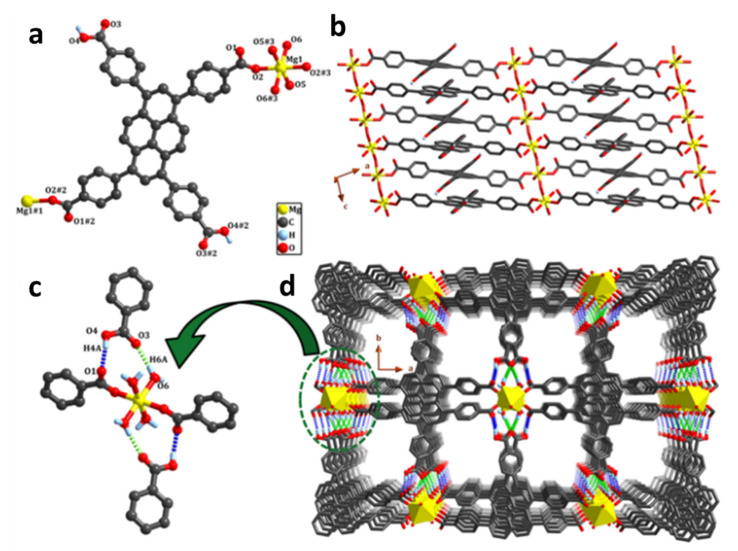
(**a**) Synergistic environment of H_2_TBAPy^2-^ and Mg^2+^. (**b**) TDL-MG 2D layer. (**c**) Strong O−H···O hydrogen bond interaction in TDL-MG. (**d**) TDL-3D MG’s supramolecular structure created via interlayer hydrogen bonding (reproduced with permission of American Chemical Society from ref. [111]).

As shown in Figure 29a, four coordinated water molecules and two distinct carboxyl oxygen atoms from H2TBAPy2 make up the center of Mg(II), which has an octahedral hexacoordinated shape [112]. The shortest 1D Mg(II)-Mg(II) chain is formed when a water molecule (O5) connects the adjacent Mg(II) centers. To create a 2D layer, these chains are linked by deprotonated H_2_TBAPy^2-^carboxylic acid groups. (Figure 29b). As illustrated in Figure 29c, the carboxyl and coordinated water molecules’ H atoms, as well as the exposed carboxyl O atoms, assist in forming the firm and directed interlayer hydrogen bindings that link the adjoining layers together to create a stable three-dimensional supramolecular structure (Figure 29d).

**Figure 30:**
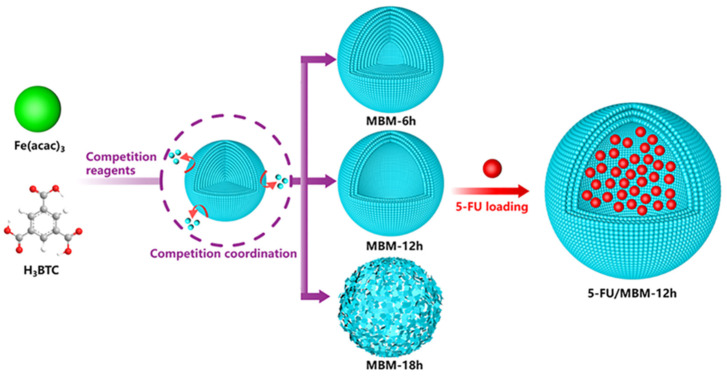
Schematic for synthesizing 5-FU/MBM-12 h microcapsules and MBM microcapsules with various reaction times (reproduced with permission of the American Chemical Society from ref. [113]).

In addition to pore encapsulation, Cui et al. prepared microcapsules (MBMs) which have characteristics of remarkable drug loading capacity, satisfactory drug release effects, and high magnetic resonance imaging capability by solvothermal method using competitive coordination method based on Fe-MOF [113]. As shown in Figure 30, the main principle is that, in order to compete with the iron site and the benzene tricarboxylic acid (BTC), ligands used to create hollow structures, polyoxometalates, and sulfides were introduced as competing agents during the synthesis of Fe-MOF. MBMs formed after 12 h of heating were selected for 5-FU loading.

In the experiment, a small amount of (NH_4_)_6_Mo_7_O_24_·4H_2_O and thiacetamide were added in the synthesis of MBMs to achieve the purpose of hollow structure competition with iron site and BTC ligand. In the subsequent experiments, 5-FU was loaded in this cavity structure, which achieved good drug loading and drug release ability. MBMs could release drugs over time without the need for special methods, and accordingly, the form of drug release over time cannot be controlled.

**Figure 31:**
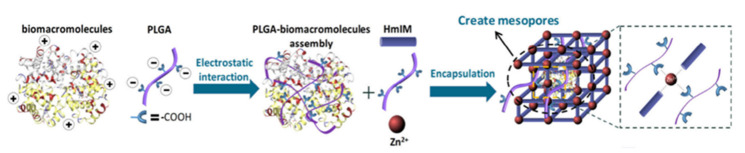
Diagram of the in situ encapsulation of biomolecules in transmutable MOFs by PLGA modulation (reproduced with permission of John Wiley & Sons, Inc. from ref. [114]).

For in situ encapsulation, Chen et al. [114] used horseradish peroxidase (HRP) as a biomacromolecule model. The MOF structure was constructed with 2-methylimidazole (HmIM) and zinc acetate to form biomacromolecules@MOFs biohybrid. However, the negative charge contributed by carboxyl groups on amino acid residues of biological macromolecules plays an important role in the process of forming MOFs around biological macromolecules. HRP with positive surface charge could not induce the formation of MOFs. Once PLGA was added, PLGA-modified HRP with many carboxyl groups would stimulate the production of biomacromolecules@MOFs complexes by electrostatic interaction (Figure 31).

**Figure 32:**
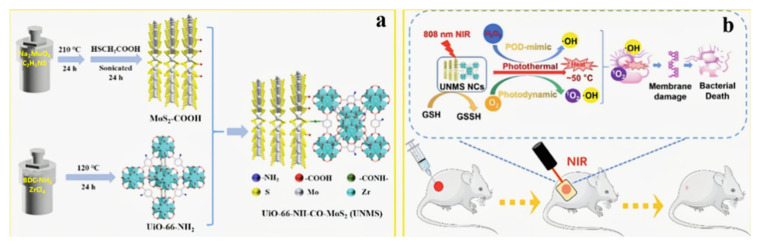
Diagram of (**a**) preparation process of UNMS NCs; (**b**) the actericidal mechanism of UNMS NCs and treatment of wound infection (reproduced with permission of John Wiley & Sons, Inc. from ref. [115]).

In addition to its use in cancer treatment, MOF can also be used to fight antibiotic resistance. Liao and co-workers used amide bonds to construct a zirconium-based MOF nanocomposite named UiO-66-NH-COMoS_2_ (UNMS NCs) (Figure 32) [115]. The antibacterial mechanism of UNMS NCs can be controlled by photothermal and photodynamic regulation of peroxidase (POD)-mimic enzymatic activity. Due to their advantages of high specific surface area and pore structure, UNMS NCs capture bacteria mainly through electrostatic interactions to improve antimicrobial efficiency. Moreover, UNMS NCs can produce good thermal and photodynamic properties under 808 nm laser irradiation. A large amount of •OH produced by high specific surface area can produce significant antibacterial effect. Additionally, 808 nm laser-induced high temperature can effectively accelerate the oxidation of glutathione (GSH) and destroy the intercellular protection system of bacteria, thus effectively improving the antibacterial efficiency against ampicillin-resistant Escherichia coli (AREC) and methicillin-resistant Staphylococcus aureus (MRSA).

### 5.2. Supramolecular Combination Therapy Based on Supramolecular Coordination Complexes

**Figure 33:**
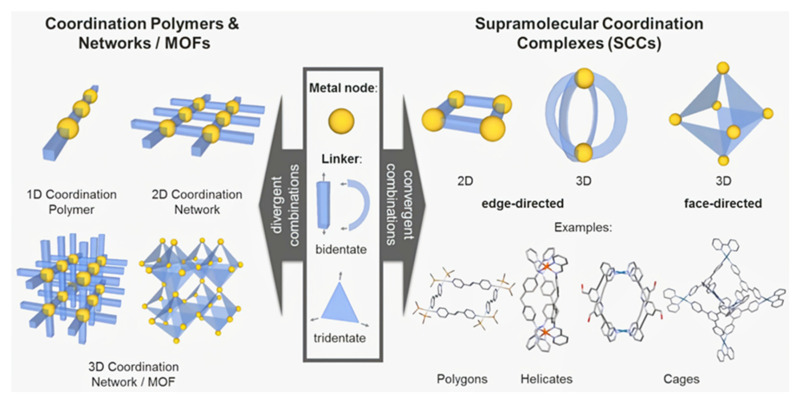
Illustration of two ways to achieve assemblies based on metal material: coordination polymers and networks produced by divergence (**left**) and discrete supramolecular complexes produced by polymeric combinations of organic linkers (**right**) (reproduced with permission of Ivyspring International Publisher from ref. [107]).

From the above statement, it is not difficult to find that the metal-to-metal connections in the MOF structure are mostly linear “short rod-like” structures and diversely arranged to form infinite coordination polymers or networks. Contrarily, discrete two-dimensional or three-dimensional frameworks can be produced if the geometric combination permits convergent arrangement of nodes and connectors, which is the supramolecular coordination complex (SCCs) mentioned above (Figure 33) [107]. The so-called edge- and face-directed approaches are two popular synthesis techniques to produce SCC [116]. The face-directed synthesis of SCC, developed by Fujita [117] and also known as the “panel method”, is constructed using planar multidentate ligand molecules that coordinate with (convergent oriented) vacancies at metal nodes to form supramolecular polyhedrons or faces of polygons. Stang et al. are credited with inventing the edge-directed synthesis technique, which uses the “banana-shaped” pyridine ligand of double-ester to form the edge of SCCs, mainly binding to metal nodes of Pd(II) and Pt(II) [118,119].

**Figure 34:**
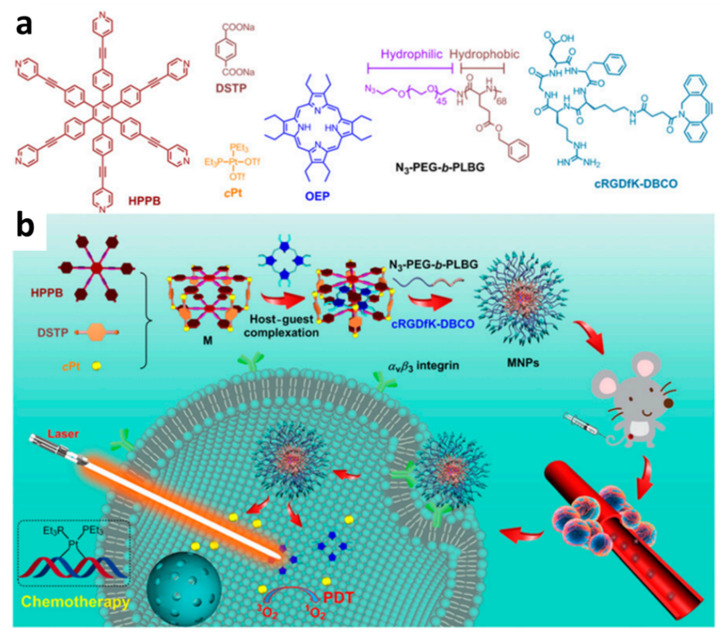
Diagram of (**a**) chemical constitution of HPPB, DSTP, cPt, OEP, N3-PEG-B-PLBG, and CrGDFK-DBCO. (**b**) The preparation and photochemical process of MNPs, as well as EPR effector and receptor-mediated tumor accumulation (reproduced with permission of National Academy of Sciences from ref. [120]).

Recently, Yu et al. used a multi-component coordination self-assembly method to prepare M from 20 molecular units of three distinct classes, 4-(4′-pyridylethynyl)phenyl (HPPB), disodium terephthalate (DSTP), and chemotherapy drugs Cis-(PEt_3_)_2_Pt(OTf)_2_ (CPT), at a ratio of 2:6:12, respectively (Figure 34) [120]. Subsequently, the host–guest compound (M@OEP) was synthesized through the host–guest interaction among M and OEP. N_3_-PEG-b-PLBG was utilized as a polymer covering to encapsulate M@OEP into its hydrophobic cavity and provide MNPs containing 30.6% M@OEP in order to boost the stability and solubility of M. The circulating RGD (cRGDfK) function was bonded to the surface of MNPs via copper-free click reaction between cRGDfK-DBCO and azide groups of the polymer shield in order to provide the nanoparticles the capacity to target cancer cells overexpressing αVβ3 integrin. Recent research has revealed that photosensitizers and chemotherapeutic drugs may be distributed to lysosomes and endosomes, indicating that photochemotherapy could act synergistically in treating tumor cells.

### 5.3. Supramolecular Combination Therapy Based on Hierarchical Self-Assembly

As mentioned above, the synthesis of SCCs adopts convergent metal binding sites to form discrete and independent coordination complexes, while MOFs adopts divergent metal binding sites to form a frame structure that can be infinitely extended. If discrete SCCs are connected in a special way, a new class of supramolecular complexes can be obtained.

**Figure 35:**
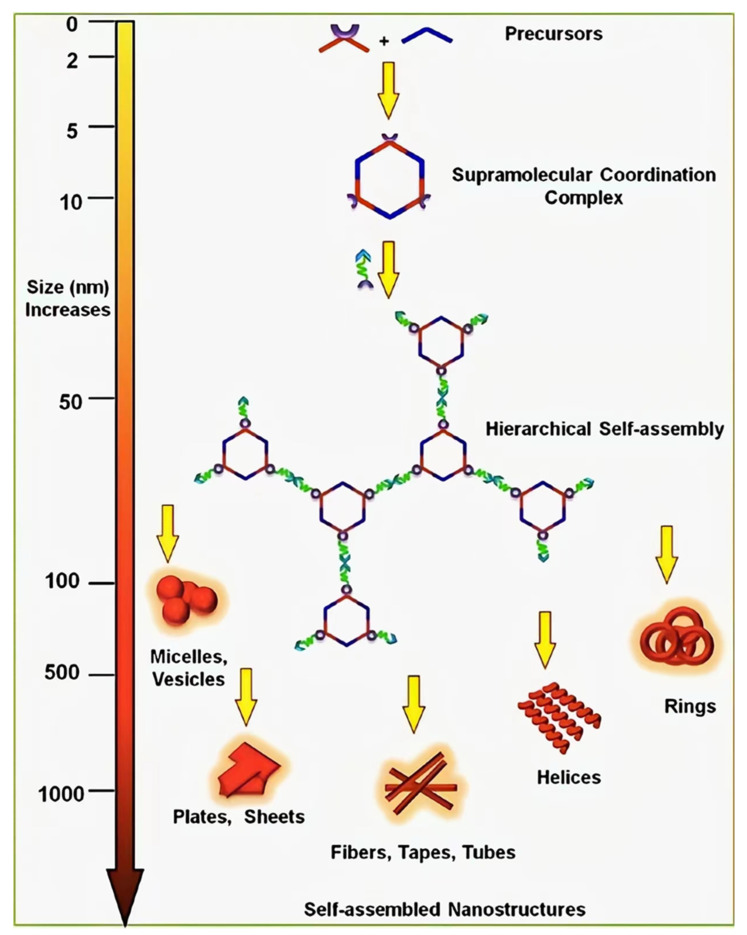
Schematic representation of hexagonal SCC prepared through coordination-driven self-assembly with suitable precursors and well-defined nanostructures of HSA assembled by it using multiple orthogonal interactions (reproduced with permission of American Chemical Society from ref. [108]).

Hierarchical self-assembly (HSA) formed through multiple non-interfering mutual effects is the foundation for the formation of varieties of complicated biotic structures, such as double-stranded DNA, 3D folded polypeptide, and bioactive cytomembranes, and is essential for cell survival [108]. Numerous noncovalent interactions, such as metal coordination, h-bonding, hydrophobic interaction, electrostatic interaction, and so on, play a significant role in the formation of HSA. When SCC is used as the original for the synthesis of HSA, metal-coordinated ligands confer considerable advantages over classical covalent methods, such as fewer steps and rapid and easy preparation of defect-free end products. The perpendicularity of metal ligands with non-covalent interactions has resulted in some unique hierarchical structures where metal acyclic cores may be built via ligand-driven self-assembly. The final hierarchy is obtained by using a second different interaction (Figure 35).

**Figure 36:**
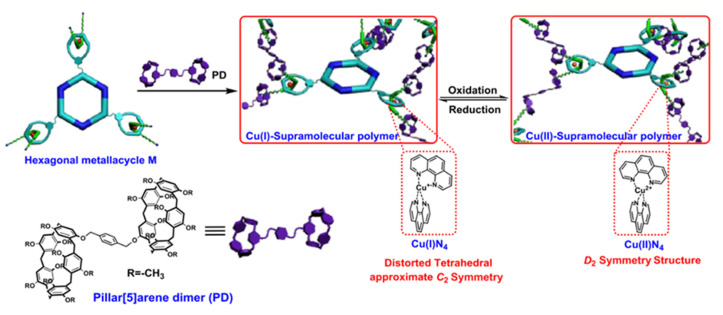
Schematic of the synthesis and redox behavior of the supramolecular polymer (reproduced with permission of American Chemical Society from ref. [121]).

Using the HSA synthesis method, Wu et al. successively synthesized two new supramolecular polymers using copper(I)-phenanthroline complexes [121,122]. In 2018, Wu et al. used a pillararene [5] dimer and a tris-[2]pseudorotaxane metallacycle containing hexacyanos on its perimeter to create a novel cross-linked supramolecular polymer. The stability of Cu(I)-[2] pseudonaphthenes can be increased by using this manufacturing method (Figure 36). The redox stimulus response behavior of the cross-linked supramolecular polymer M@(PD)3 causes a reversible modification of the supramolecular polymer’s diffusion coefficient by altering the redox state of the metal complexes.

**Figure 37:**
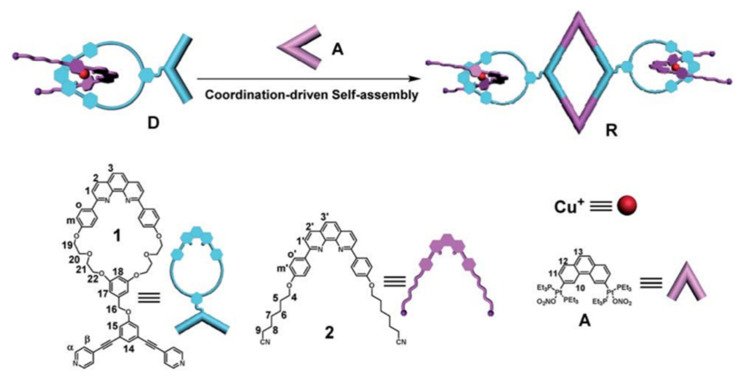
Illustration of the self-assembly bis-[2]pseudorotaxanes rhombohedral metallacycle (reproduced with permission of the Royal Society of Chemistry from ref. [122]).

Recently, on the basis of the above studies, Wu et al. created another redox supramolecular polymer using a well-defined rhomboidal bis-[2]pseudorotaxanes metallacycle based on coordination-driven self-assembly and host–guest interaction employing hierarchical orthogonal design strategy using Cu(I)-phenanthroline complex as a new host for linkage (Figure 37). In the supramolecular polymer produced by this scheme, the host–guest interaction was like the covalent bond end sealing process, and reactive redox stimulation used Cu(I)N4[2] pseudonaphthenes as the active site. The unstable tetrahedral complex Cu(II)N4 may be able to retain sufficient stability in redox processes with the aid of supramolecular polymerization. The first step in the complex preparation process is to construct [2]pseudonaphthenes with a dipyridinium (120°) donor D using a transitional Cu(I) template strategy consisting of a 120° macroloop 1 containing a fenyl and ligand 2 with a cyanosite. Subsequently, Cu(I) was used as template for the threading process of D. Finally, in order to create the novel bis-[2]pseudorotaxanes rhombohedral metallacycle R, donor building block D and matching 60° receiver A were mixed in 1 and self-assembled by coordination drive. Compared to the 2017 study, the rhombohedral metallacycle has a smaller skeleton size than the hexagonal metallacycle M, which was previously assembled using a 120° receptor. Based on the theories of the above two studies, the prepared polymers can be stimulated by redox reactions to achieve drug release when used for drug delivery.

Combined with the above narration, it is not difficult to find that cyclodextrin, pillararenes, and cucurbiturils have similar macrocyclic structures. When they are applied to construct drug delivery systems, if drugs are placed in their cavities, the differences among the three are mainly reflected in the size of the cavities, which affects the selection of drug molecules to a certain extent. They bind to the guest molecule with a non-covalent bond. Cyclodextrins mainly use hydrophobic interaction and van der Waals forces to form stable structures; pillararenes use hydrophobic interaction, hydrogen bonds, and electrostatic interactions to form stable structures; cucurbiturils use hydrogen bonds, van der waals forces, and the interaction between ion dipoles and CB[n] channels to form stable structures [21]. Because these three have the function of transforming the optical properties of drugs, they show excellent advantages in the combination therapy involving phototherapy. The advantages of metal coordination schemes over macrocyclic schemes are mainly reflected in their high programmability. The strong directionality between the metal and the ligand drives the overall structure of the complex via self-assembly. Moreover, a variety of metal elements may show different effects on different treatment regimens when applied to drug delivery.

## 6. Summary and Outlook

The combination cancer therapies of supramolecular macrocyclic materials have been summarized in this review. We introduce different combination cancer therapy based on cyclodextrin, pillararene, and cucurbituril through host–guest interaction. The design and development of macrocyclic supramolecular drug delivery system is a fascinating project. The binding affinity of macrocyclic supramolecular materials and chemotherapeutic drugs are identified by ITC techniques. Interestingly, macrocyclic supramolecular cancer chemotherapy has exhibited synergistic anti-cancer effects to combine with photothermal, gene, radiation, and protein therapy.

The design of supramolecular combination cancer therapy is an intriguing project. However, the application is in its infancy. The relevant issues between supramolecular structure and functions in human biological environment and application in clinics still exist. It is anticipated that this review will enrich practical application of the macrocyclic supramolecular materials for cancer therapy in the wider picture.

## Data Availability

Not applicable.
